# Transcriptome and Metabolome Analyses Revealed the Response Mechanism of Quinoa Seedlings to Different Phosphorus Stresses

**DOI:** 10.3390/ijms23094704

**Published:** 2022-04-24

**Authors:** Qianchao Wang, Yirui Guo, Tingzhi Huang, Xuesong Zhang, Ping Zhang, Heng Xie, Junna Liu, Li Li, Zhiyou Kong, Peng Qin

**Affiliations:** 1 College of Agronomy and Biotechnology, Yunnan Agricultural University, Kunming 650201, China; 2020110028@stu.ynau.edu.cn (Q.W.); 2020240137@stu.ynau.edu.cn (Y.G.); 2020240162@stu.ynau.edu.cn (T.H.); 2020240160@stu.ynau.edu.cn (X.Z.); 2021110031@stu.ynau.edu.cn (P.Z.); 2020210159@stu.ynau.edu.cn (H.X.); 2021110026@stu.ynau.edu.cn (J.L.); 2019210130@stu.ynau.edu.cn (L.L.); 2 College of Natural Resources and Environment, Baoshan University, Baoshan 678000, China

**Keywords:** metabolome, phosphorus level, quinoa, transcriptome

## Abstract

Quinoa (*Chenopodium quinoa* Willd.) is a dicotyledonous annual herb of Family Amaranthaceae and Subfamily Chenopodiaceae. It has high nutritional and economic value. Phosphorus (P) is an essential plant macronutrient, a component of many biomolecules, and vital to growth, development, and metabolism. We analyzed the transcriptomes and metabolomes of Dianli–1299 and Dianli–71 quinoa seedlings, compared their phenotypes, and elucidated the mechanisms of their responses to the phosphorus treatments. Phenotypes significantly varied with phosphorus level. The plants responded to changes in available phosphorus by modulating metabolites and genes implicated in glycerophospholipid, glycerolipid and glycolysis, and glyconeogenesis metabolism. We detected 1057 metabolites, of which 149 were differentially expressed (DEMs) and common to the control (CK) vs. the low-phosphorus (LP) treatment samples, while two DEMs were common to CK vs. the high-phosphorus (HP) treatment samples. The Kyoto Encyclopedia of genes and genomes (KEGG) annotated 29,232 genes, of which 231 were differentially expressed (DEGs) and common to CK vs. LP, while one was common to CK vs. HP. A total of 15 DEMs and 11 DEGs might account for the observed differences in the responses of the quinoa seedlings to the various phosphorus levels. The foregoing results may provide a theoretical basis for improving the phosphorus utilization efficiency in quinoa.

## 1. Introduction

Quinoa (*Chenopodium quinoa* Willd.) is an annual self-pollinated dicotyledonous herbaceous crop in Subfamily Chenopodiaceae of Family Amaranthaceae [1,2]. It is also known as Indian wheat, gray rice, and golden cereal. It is native to the Andes Mountains in South America, where it grows at 2800–5000 m a.s.l. Cold, drought, nutrient deficiency, and salinity tolerance are characteristics of high-quality quinoa varieties [3]. Quinoa seeds are abundant in proteins, amino acids, polyphenols, vitamins, flavonoids, unsaturated fatty acids, and other components and meet the basic nutritional requirements of humans [4,5,6]. Quinoa has antioxidant, anti-aging, hypolipidemic, hypoglycemic, and hypotensive effects prevents cardiovascular and cerebrovascular diseases and may reduce the risk of certain chronic disorders [2,7,8]. Therefore, quinoa has been recognized by the Food and Agriculture Organization of the United Nations (FAO) as the only monocrop that meets all nutritional needs of the human body [9]. It was also designated one of the food security crops of the century [10]. International nutritionists call it a “supergrain” and recommend it as the perfect “whole food” [11].

Phosphorus (P) is an essential element for plant growth and development. It is a major constituent of several vital biomolecules and plays important roles in plant metabolism [12]. Proper phosphorus fertilizer application improves crop yield and quality [13]. In ~46% of the global cropland, phosphorus use efficiency is inadequate [14]. Moreover, most phosphorus is converted to other forms that plants can neither absorb nor assimilate. Hence, the phosphorus is eventually lost to the environment [15]. Phosphate fertilizer consumption is steadily increasing and is expected to continue rising in the future. However, bioavailable phosphorus is limited in the soil and may be insufficient to meet plant growth and developmental requirements. Hence, phosphorus shortages and price increases are anticipated and could impede progress in global agriculture [16]. Plant species differ in terms of their phosphorus needs. Therefore, it is imperative to explore the effects of phosphorus content on plant growth and development.

Under low-phosphorus conditions, soluble phosphorus is released by changing or replacing the biofilm structure and composition and maintaining basic plant growth and development [17,18,19]. When there is too much phosphorus, it can enhance the respiration of plants, consume a large amount of carbon hydration platforms, have thick and dense leaves, cause premature development of reproductive organs, inhibition of stem and leaf growth, cessation of vegetative growth and excessive prematurely, resulting in reduced yield. Various lipids constitute the cell membrane. Plant growth, development, and physiological and biochemical functions affect membrane lipid structure and composition [20]. The cell membrane is mainly composed of lipids (mainly phospholipids), proteins, and sugars. The cell membrane is semipermeable and comprises mainly phospholipids. Sphingolipids, sterols, and glycerolipids are principal components of plant cell membranes [21]. In higher plants, the phospholipids include phosphatidylcholine (PC), phosphatidylserine (PS), phosphatidylethanolamine (PE), phosphatidylinositol (PI), phosphatidylic acid (PA), and phosphatidylglycerol (PG) [19,20,21]. Monogalactosyldiacylglycerol (MGDG), sulfoquinovosyldiacylglycerol (SQDG), and digalactosyldiacylglycerol (DGDG) constitute the glycolipids [19]. Under abiotic stress, plants could secrete several metabolites and regulate some genes to stabilize their intracellular environment. Cheng et al. [22] conducted a genome-wide association study (GWAS) on two natural plant populations and detected 259 candidate genes that respond to low-phosphorus stress. Xu et al. [23] found that in response to phosphorus deficiency, LaABCG36s and LaABCG37s mediate phytohormone activity, which, in turn, promotes clumping root formation and improves low-phosphorus tolerance in white lupin. Wang et al. [24] reported that oats presented with significantly elevated root citric and malic acid levels and upregulation of 48 related genes after ~1 mo of low-phosphorus stress. Ting et al. [25] stated that variations in the phosphorus levels influenced the phenotypic characteristics and flavonoid and anthocyanin content in apple roots and leaves. Zangani E et al. [26] reported that proper application of phosphorus fertilizer could affect leaf stomatal conductance, photosynthetic response, and seed yield of rapeseed.

Numerous recent studies have reported on the effects of nitrogen and potassium fertilizer on quinoa growth and development. By contrast, few reports have been published on the impact of phosphorus fertilizer on quinoa seedling growth and development. Here, we used ultra-high performance liquid chromatography and tandem mass spectrometry (UPLC-MS/MS) to evaluate the effects of different phosphorus levels on quinoa seedling metabolites. We also used transcriptomics to screen DEGs in quinoa seedlings subjected to various phosphorus levels. We used the foregoing information and morphological indices to elucidate the response mechanisms of quinoa seedlings to different phosphorus levels. The results of this study may provide a theoretical basis for molecular breeding, rational and scientific fertilization, and standardized production of high-yield, high-quality quinoa.

## 2. Results

### 2.1. Agronomic Characteristics of Quinoa Seedlings under Different Phosphorus Levels

**Figure 1:**
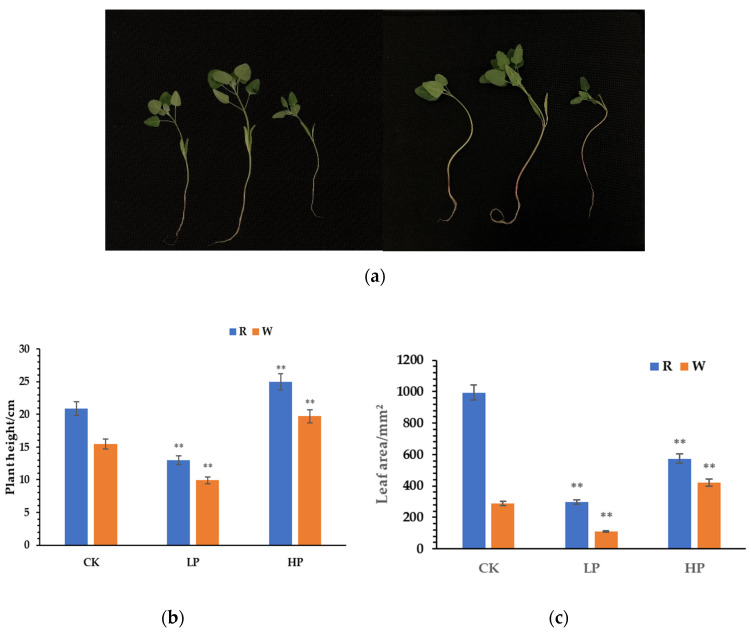
(**a**) Relative plant growth (from left to right) for red and white quinoa CK, HP, and LP. (**b**) CK, LP, and HP seedling heights. (**c**) CK, LP, and HP leaf areas. ‘R’ represents red quinoa, ‘W’ represents white quinoa; ** indicates that the seedling heights and leaf areas of each variety under various phosphorus concentrations were significantly different from those of the control condition at *p* < 0.01 (Student’s *t*-test).

Quinoa seedling morphology significantly differed after 30 d under the various phosphorus concentrations (Figure 1a). The order of plant height was HP > CK > LP, and the differences were significant (Figure 1b). For red quinoa, the order of leaf area was HP ≅ CK > LP, while for white quinoa, it was HP > CK > LP (Figure 1c), and the differences showed that the plant heights and leaf areas of quinoa seedlings increased with the increase of phosphorus content.

### 2.2. Qualitative and Quantitative Analyses of Metabolites in Quinoa Seedling under Different Phosphorus Levels

**Figure 2:**
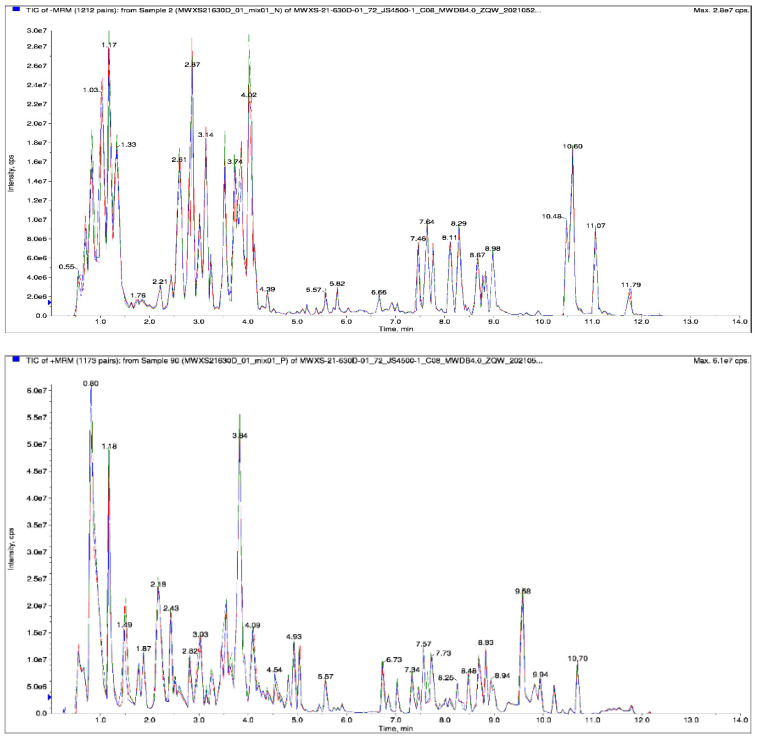
Total ion flow (TIC) showing positive and negative ion modes (from (**top**) to (**bottom**)).

**Figure 3:**
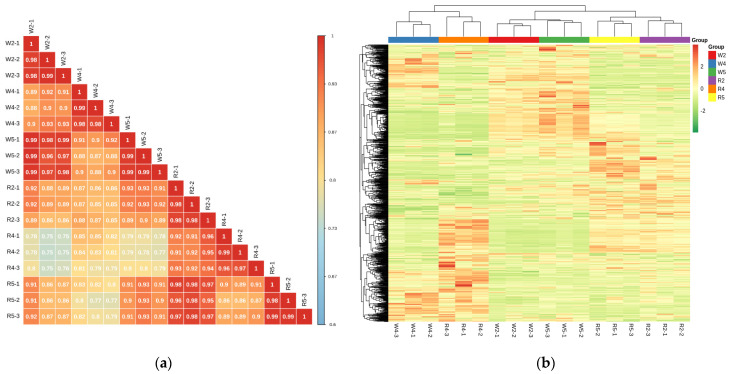
(**a**) Correlations among samples. (**b**) Overall cluster analysis heat map of samples.

For the metabolomics study, 18 samples were selected and divided into 6 groups. There were three biological replicates per group. The metabolites were quantified by triple-quadrupole mass spectrometry (QQQ-MS) in the multiple reaction monitoring mode (MRM) (Figure S1) with high accuracy and good repeatability. Overlapping the display of the total ion flow (TIC) plots for MS detection and analysis of various QC samples showed a high curve overlap of the total ion flow detected by the metabolites. The retention times and peak intensities were consistent. Hence, the MS signal was stable when the same samples were detected at different times (Figure 2), indicating good instrument stability and data reliability. The correlation diagrams (Figure 3a) and the overall cluster analysis heat maps of the samples (Figure 3b) demonstrated good biological repeatability within sample groups and significant differences between groups in terms of their related metabolites.

**Figure 4:**
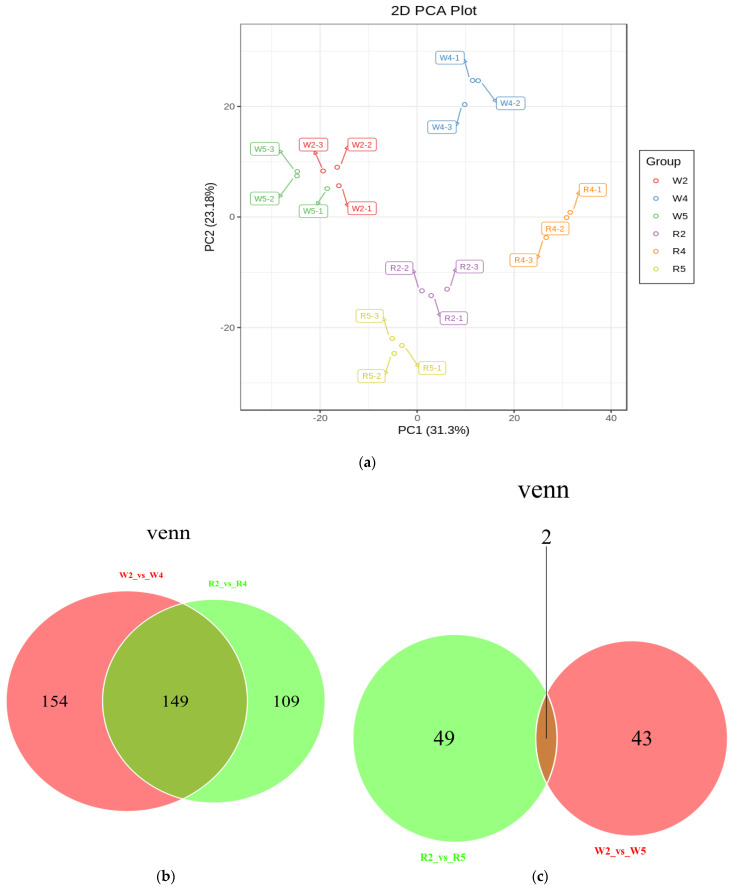

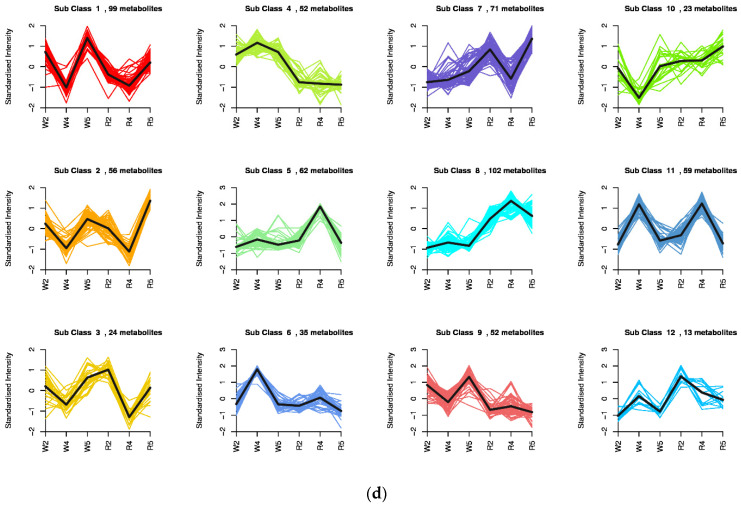
(**a**) PCA score chart of MS data for all QC samples and sample groups. (**b**) Venn diagram of DEMs in multiple pairwise comparisons of CK vs. LP. (**c**) Venn diagram of DEMs in multiple pairwise comparison of CK vs. HP. Each circle represents a comparator group, numbers in ellipse overlaps represent the number of DEMs common to the comparator group, and numbers outside overlaps represent the number of specific differential metabolites of the comparator group. (**d**) K-means diagram of DEMs. The X-coordinate represents the sample. The Y-coordinate represents the relative standardized metabolite content.

Principal component analysis (PCA) can preliminarily understand the overall metabolic difference between each group and the degree of variability between samples. PCA score chart (Figure 4a) and grouping principal component analysis chart (Figure S2), and an orthogonal partial least squares discriminant analysis (OPLS-DA) score chart (Figure S3) were performed on all samples and disclosed significant differences among LP, HP, and CK at different phosphorus levels. In order to effectively deal with the variables with small correlation, the OPLS-DA model was run on the data in 200 randomized permutation experiments (Figure S4). All Q^2^ were >0.9 except for R2 vs. R5 (Q^2^ = 0.815) and W2 vs. W5 (Q^2^ = 0.746). For all models, *p* was < 0.05. Therefore, the OPLS-DA model had reliable predictability. Based on the OPLS-DA results, metabolites with a fold change of ≥2 or ≤0.5 were selected for the differential analysis. There were 149 common DEMs in CK vs. LP (Figure 4b; Table S1) and two common DEMs in CK vs. HP (Figure 4c; Table S2).

The relative contents of the DEMs were standardized and centralized, and then a K-means clustering analysis was carried out. The DEMs were divided into 12 classes by K-means clustering analysis (Figure 4d; Table S3). The clusters of LP were significantly lower than the first, second, and third clusters but significantly higher than the fifth, sixth, eighth, and eleventh clusters of CK. The clusters of HP were significantly higher than the first, second, and seventh clusters of CK. Among them, the metabolites in clusters 4, 5, 6, 8, 9, and 11 were significantly higher in CK vs. HP than CK vs. LP. Hence, the sequencing data quality was high. Based on the KEGG compound database, the MetWare database (MWDB), and MRM, 1057 metabolites were detected, including 98 amino acids and their derivatives, 166 phenolic acids, 69 nucleotides and their derivatives, 172 flavonoids, 11 quinones, 24 lignins and coumarins, 9 tannins, 78 alkaloids, 39 terpenoids, 94 organic acids, 177 lipids, and 120 compounds in other chemical classes (Table S4).

### 2.3. Differentially Expressed Metabolite Analysis and Enrichment

For the various phosphorus levels, 1057 DEMs were screened by combining variable importance projections (VIP) (Figure S5) and fold changes. Different metabolites interact in organisms to form different pathways. According to the KEGG pathway database, the enrichment analysis of the KEGG pathway was conducted, and pathways with *p* ≤ 0.05 were selected. The significantly enriched pathways were Carbon fixation in photosynthetic organisms, Starch and sucrose metabolism, Purine metabolism, Glycolysis/Gluconeogenesis, Glycerolipid metabolism, and Glycerophospholipid metabolism. For CK vs. LP, the common metabolites under Glycerolipid metabolism were dihydroxyacetone phosphate, 3-phospho-*D*-glyceric acid, glucose-1-phosphate *, and uridine 5′-diphospho-*D*-glucose, and all of them were downregulated. The major DEMs under Glycerolipid metabolism in quinoa seedlings subjected to the various phosphorus levels were nucleotides and their derivatives (Table S5). There were five DEMs in the Glycolysis/Gluconeogenesis pathway of CK vs. LP, namely, phosphoenolpyruvate (PEP), dihydroxyacetone phosphate, 3-phospho-*D*-glyceric acid, glucose-1-phosphate *, and salicin. The last was upregulated, while the first four were downregulated in CK vs. LP. PEP, 3-phospho-*D*-glyceric acid, and glucose-1-phosphate * were upregulated in red quinoa with R2 vs. R4. Salidroside downregulation under R2 and R4 indicated that saccharides, alcohols, and phenolic acids were the principal DEMs in the Glycolysis/Gluconeogenesis pathway of quinoa seedlings subjected to different phosphorus levels (Table S6).

### 2.4. Transcriptome Analysis of Quinoa Seedlings Subjected to Different Phosphorus Levels

**Figure 5:**
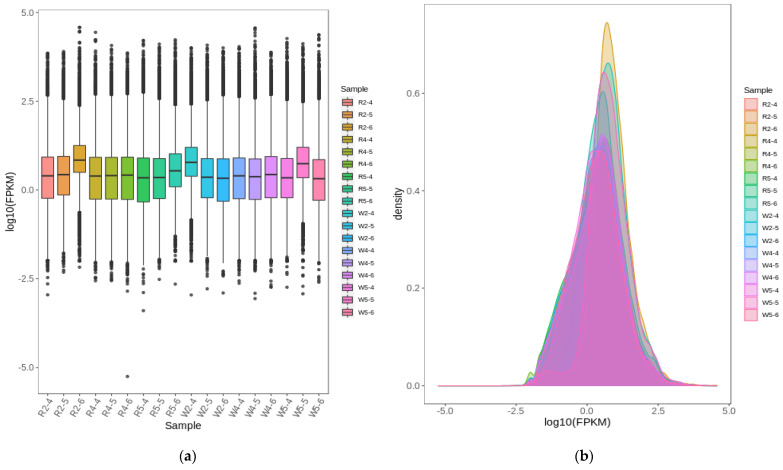

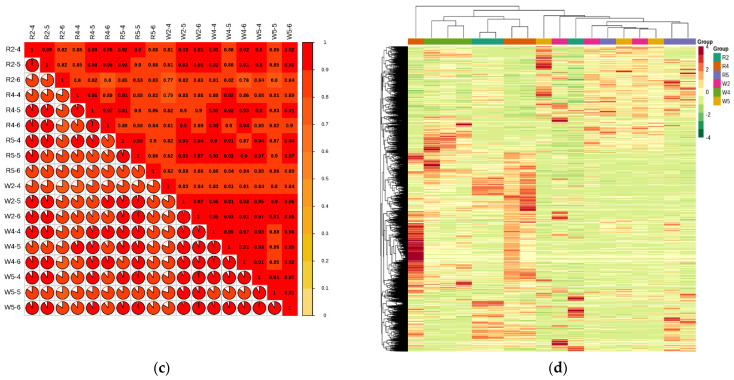
(**a**) Gene expression box line plot. Abscissa indicates different samples. Ordinate indicates logarithmic values of sample expression FPKM. The graph measures the distribution of overall gene expression levels for each sample. (**b**) Expression density distribution plot. Different colors of curves in the graph represent different samples. Abscissa of points on the curve indicates logarithmic values of FPKM for corresponding samples. Ordinate of points on the curve indicates probability density. (**c**) Correlation heat map. Pearson’s correlation coefficient (R^2^) > 0.8 between biological replicate samples. (**d**) Cluster heat map. Abscissa indicates sample names and hierarchical clustering results. Ordinate indicates DEGs and hierarchical clustering results. Red and green indicate high and low expression, respectively.

Transcriptome sequencing was performed using aboveground parts of the two quinoa strains, with three biological replicates each. After filtering the original data, determining the sequencing error rate, and establishing the GC content distribution, 139.76 Gb of clean data was obtained. For each sample, there were 6 Gb clean data and a Q30 score of at least 91% (Table S7). The proportion of sequenced reads successfully compared against the genome was >70%, and the comparison efficiency was >80%. Thus, the sequencing was highly accurate, indicating that the transcriptome data met the requirements for subsequent analysis. The FPKM (fragments per kilobase of transcript per million fragments mapped) box line plot of the sample distribution is shown in Figure 5a. The dispersion of gene expression level distribution per sample was small, and the overall gene expression was high. The expression density of the distribution plot reveals that the gene abundance was relatively more concentrated where changes in gene expression level occurred at the different phosphorus concentrations. The gene expression FPKM concentration range was 10^−2.5^–10^2.5^ (Figure 5b). The correlation heat map (Figure 5c) demonstrates a high degree of biological reproducibility among samples. Here, R^2^ > 0.8 between biological replicates subjected to the different phosphorus levels. Extracting the FPKM expression after centralization and normalization of differential genes, doing hierarchical cluster analysis, and plotting the cluster heat map for each differential grouping showed that there were significant differences in gene expression at different phosphorus levels in this experiment (Figure 5d). In conclusion, it is ready for further searching for differentially expressed genes.

### 2.5. Functional Annotation and Enrichment Analysis of Differentially Expressed Genes

KEGG, GO, NR, Swiss-Prot, KOG, Pfam, and TrEMBL were functionally annotated to 29,232, 41,046, 53,275, 34,131, 48,791, 42,701, and 51,060 genes, respectively.

**Figure 6:**
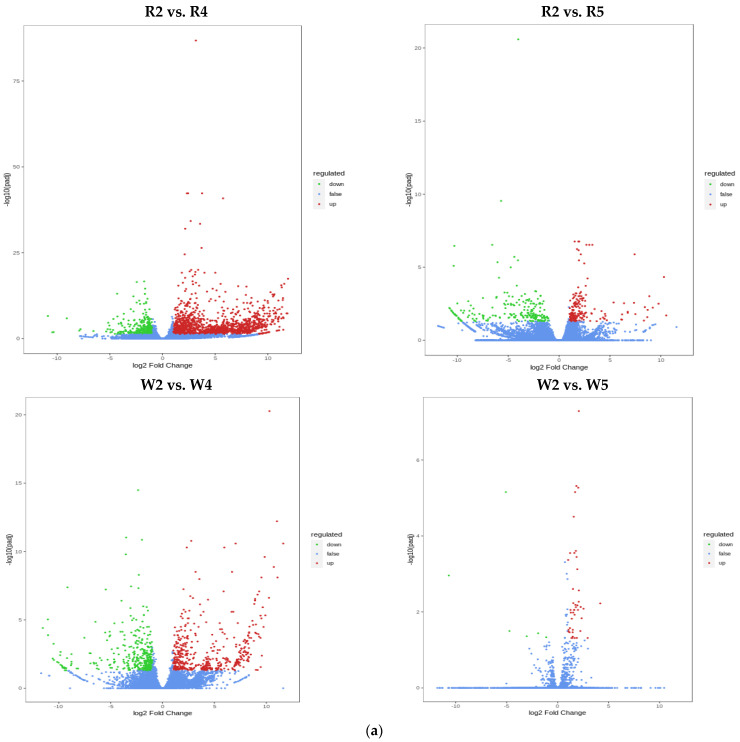

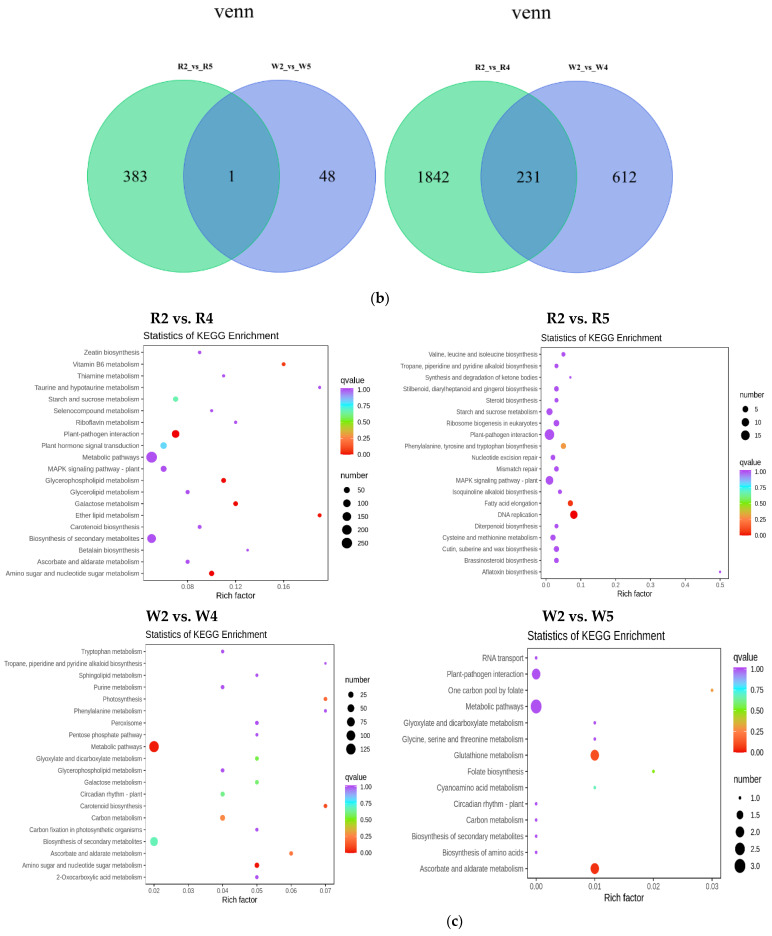
(**a**) Volcano map of DEGs; R2 vs. R4, R2 vs. R5, W2 vs. W4, and W2 vs. W5 (from left to right). Abscissa represents multiples of changes in gene expression. Ordinate represents DEGs significance levels. Red and green dots represent upregulated and downregulated DEGs, respectively. Blue dots represent non-DEGs. (**b**) Venn diagram of DEGs; CK vs. LP and CK vs. HP. (**c**) Enrichment scatter diagram; R2 vs. R4, R2 vs. R5, W2 vs. W4, and W2 vs. W5 (from left to right). The ordinate and abscissa represent the KEGG pathway and rich factor, respectively. The degree of enrichment increases with rich factor. The dot size is proportional to the number of enriched DEGs in the pathway. Red color intensity increases with enrichment significance.

The differentially expressed genes (DEGs) were analyzed with DESeq2, and the total, upregulated, and downregulated DEGs per group were enumerated (Table 1). A volcano map shows the overall distribution of the DEGs in both sample groups (Figure 6a). The FPKM of DEGs was centralized and normalized, and a K-means clustering analysis was performed. The objectives were to clarify the regulatory patterns of the genes in the quinoa seedlings subjected to different phosphorus levels and identify similar change trends within the same functional gene classes under different experimental treatments. Clusters 3, 4, and 7 exhibited the same expression trends under low phosphorus levels. Hence, they could serve as markers distinguishing gene expression in phosphorus deficiency (Figure S6), extract the expression of FPKM after centralization and standardization of differential genes, perform hierarchical clustering analysis, and draw the clustering heat map of each differential group (Figure S7). Hierarchical clustering revealed that DEGs differed due to the phosphorus level. Venn analysis revealed 231 and 1 common DEGs for CK vs. LP and CK vs. HP, respectively (Figure 6b). The KEGG enrichment analysis output was plotted as a scatter diagram (Figure 6c), in which the enrichment degree of KEGG was determined based on the rich factor, Q-value, and the number of genes in a given pathway. The DEGs were mainly enriched in Metabolic pathways, Biosynthesis of secondary metabolites, Starch and sucrose metabolism, Glycerophospholipid metabolism, Glycerolipid metabolism, Glycolysis/Glyconeogenesis, and Amino sugar and nucleotide sugar metabolism. For each comparison, the 20 most significantly enriched pathway entries are shown (Figure 6c) or all of them if less than 20 pathway entries were enriched.

### 2.6. Combined Transcriptome and Metabolomics Analyses of the Response Mechanisms of Quinoa Seedlings to Different Phosphorus Levels

**Figure 7:**
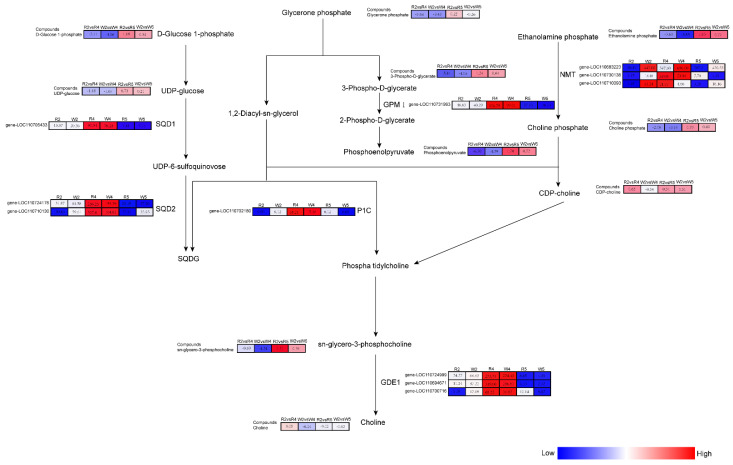
Response mechanisms of the Glycerophospholipid metabolism, Glycerolipid metabolism, and Glycolysis/Glyconeogenesis pathways in quinoa seedlings subjected to various phosphorus levels. The FPKM values indicate the gene expression levels. The log2fc values between comparisons indicate the metabolite expression levels. DEGs and DEMs are represented by boxes in pathways. Red and blue represent gene expression levels and metabolite upregulation and downregulation, respectively. SQD1, UDP-sulfoquinovose synthase; SQD2, sulfoquinovosyltransferase; GPMⅠ, 2,3-bisphosphoglycerate-independent phosphoglycerate mutase; P1C, phospholipase C; GDE1, glycerophosphodiester phosphodiesterase; NMT, phosphoethanolamine *N*-methyltransferase.

The metabolome and transcriptome data were integrated to elucidate the mechanisms by which quinoa seedlings respond to different phosphorus levels. Based on the DEM and DEG enrichment analyses, a histogram was plotted to show the extent of pathway enrichment associated with the DEMs and DEGs. The Glycerophospholipid metabolism, Glycerolipid metabolism, and Glycolysis/Glyconeogenesis pathways were significantly enriched for CK vs. LP (Figure S8). The genes and metabolites in each group with Pearson’s correlation coefficients >0.8 are shown in a nine-quadrant diagram and categorized as: (1) neither genes nor metabolites differentially expressed; (2) genes and metabolites differentially expressed in the same pattern; and (3) genes and metabolites differentially expressed in opposite patterns (Figure S9). Figure 7 shows the annotated DEMs and DEGs for the Glycerophospholipid metabolism, Glycerolipid metabolism, and Glycolysis/Glyconeogenesis pathways in the quinoa seedlings subjected to the different phosphorus levels.

In the Glycolysis/Glyconeogenesis pathway, glycerone phosphate, 3-phospho-*D*-glycerate, and PEP were significantly downregulated in CK vs. LP but significantly upregulated in CK vs. HP. In the Glycerolipid metabolism pathway, glycerone phosphate, UDP-glucose, and *D*-glucose 1-phosphate were significantly downregulated in CK vs. LP and significantly upregulated in CK vs. HP. In the Glycerophospholipid metabolism pathway, glycerone phosphate, sn-glycero-3-phosphocholine, choline phosphate, and ethanolamine phosphate were significantly downregulated in CK vs. LP and significantly upregulated in CK vs. HP.

In CK vs. LP, 2,3-bisphosphoglycerate-independent phosphoglycerate mutase [EC:5.4.2.12] (gene-LOC110731993, GPMⅠ), UDP-sulfoquinovose synthase [EC:3.13.1.1] (gene-LOC110705433, SQD1), sulfoquinovosyltransferase [EC:2.4.1.-] (gene-LOC110710130, gene-LOC110724176, SQD2), glycerophosphodiester phosphodiesterase [EC:3.1.4.46] (gene-LOC110724999, gene-LOC110694671, gene-LOC110700716, GDE1), phospholipase C [EC:3.1.4.3] (gene-LOC110702180, P1C), and phosphoethanolamine N-methyltransferase [E2.1.1.103] (gene-LOC110683223, gene-LOC110730138, NMT) were significantly upregulated. However, phosphoethanolamine *N*-methyltransferase [EC:2.1.1.103] (gene-LOC110710093, NMT) and glycerophosphodiester phosphodiesterase [EC:3.1.4.46] (gene-LOC110724999, GDE1) were significantly downregulated in response to HP stress. Choline phosphate accumulation was affected by phosphoethanolamine *N*-methyltransferase [E2.1.1.103] (gene-LOC110683223, gene-LOC110730138, gene-LOC110710093, NMT). Choline accumulation was affected by glycerophosphodiester phosphodiesterase [EC:3.1.4.46] (gene-LOC110724999, GDE1). 2-Phospho-*D*-glycerate was affected by 2,3-bisphosphoglycerate-independent phosphoglycerate mutase [EC:5.4.2.12] (gene-LOC110731993, GPMⅠ). UDP-glucose affected SQDG accumulation via UDP-sulfoquinovose synthase [EC:3.13.1.1] (gene-LOC110705433, SQD1) and sulfoquinovosyltransferase [EC:2.4.1.-] (gene-LOC110710130, gene-LOC110724176, SQD2) (Figure 7). In general, these 15 DEMs and 11 DEGs may be the key factors for quinoa seedlings to cope with different phosphorus levels.

RNA-Seq analysis and reverse-transcription PCR were performed on randomly selected DEGs to determine the authenticity and reliability of the transcriptome data and differential expression of the candidate genes. The RT-qPCR and RNA-Seq results were consistent for nine of the ten validated genes (gene-LOC110694671, gene-LOC110705433, gene-LOC110708023, gene-LOC110724999, gene-LOC110725562, gene-LOC110702180, gene-LOC110736814, gene-LOC110682517, and gene-LOC110685928). Hence, the transcriptome sequencing was reliable (Figure S10).

## 3. Discussion

Quinoa is rich in various amino acids, proteins, vitamins, and lipids vital to human health [27]. Phosphorus is an essential plant macronutrient and is integrated into nucleic acids, nucleotides, coenzymes, phospholipids, phytic acid, and so on. Phosphorus plays a key role in ATP-mediated reactions and carbohydrate, protein, and lipid metabolism. Plant morphological performance may vary with phosphorus level. At low phosphorus concentrations, plant height, shoot and root dry weight, and total root length, were significantly reduced relative to the control [28]. Similar findings were recorded in the present study as well.

Here, the LP quinoa seedlings stopped growing while the CK and HP quinoa seedlings continued to grow. However, the growth rate of HP was higher than that of CK. The LP plants were the shortest and had the smallest leaf areas. By contrast, the HP seedlings were the tallest and had the largest leaf areas. Hence, quinoa growth substantially varied with the phosphorus content. Excessively high phosphorus levels can be phytotoxic, alter root-to-shoot ratios, and lower both crop yield and quality [29]. In the present study, the HP quinoa seedlings grew best and had the optimal phenotypic state. Therefore, the maximum phosphorus dosage used here was evidently not high enough to cause phytotoxicity in the quinoa seedlings. For this reason, even higher phosphorus concentrations could be tested in future experiments; perhaps subsequent experiments could quantitate the impact of varying phosphate fertilization on quinoa grain yield and quality as well. Under phosphorus-deficient conditions, certain crops mobilize endogenous phosphorus from phospholipids rather than metabolizing glycolipids. At low phosphorus levels, plant biofilm structure and composition change to a certain extent and may solubilize and release phosphorus required for plant growth, development, and metabolism [17,18,19]. SQDG is an acidic lipid. It widely exists in plant chloroplasts and participates in the function and evolution of photosynthetic membranes. It is of great significance for plant photosynthesis [30]. The MGDG content significantly changes in rice seedlings subjected to low-phosphorus conditions [31]. UDP-glucose is a nucleotide sugar that donates glucose residues in various glycosylation reactions [32]. It is required for cytoplasmic sucrose formation and the synthesis of substances such as cellulose in the plastid extracellular body. It is also indirectly involved in hemicellulose and pectin formation in cell walls [32,33,34,35,36,37,38]. It donates glucose for the synthesis of the carbohydrate moieties of sulfonyl lipids, glycoproteins, and glycolipids and is essential in different glycosylation metabolic processes [39]. The sulfolipid sulfoquinosyldialdiallycerol is a natural sulfonic acid and a component of plant photosynthetic membranes [32]. In sulfolipid biosynthesis, sulfoquinovose (6-deoxy-6-sulfoglucose) is transferred from UDP-sulfoquinovose to diacylglycerol. UDP-glucose plus a sulfur donor form UDP-sulfoquinovose. UDP-glucose and a sulfur donor form UDP-sulfoquinovose catalyzed by bacterial SQDB protein or homologous plant SQD1 protein [19,32]. In quinoa, UDP-sulfoquinovose production is catalyzed by SQD1. Here, low-phosphorus stress significantly reduced the *D*-glucose-1-phosphate and UDP-glucose content in the quinoa seedlings. Consequently, photosynthesis was attenuated, the plants were short, and the leaves were small. In contrast, the HP seedlings presented with significantly higher levels of *D*-glucose-1-phosphate and UDP-glucose. Essigmann [40] and Sandaet [32] reported similar findings. Nucleic acids, nucleoproteins, and phospholipids have absolute phosphorus requirements and are vital for plant growth and development. In plants, phosphorus an plays important role in cell division and growth, carbohydrate and lipid metabolism, and amino acid, protein, and carbohydrate synthesis and transport. We found that when the quinoa seedlings were subjected to low phosphorus levels, their glycerone phosphate content was significantly reduced and was followed by corresponding reductions in 3-phospho-*D*-glycerate, sn-glycero-3-phosphocholine, and ethanolamine phosphate, lowered cell viability, and slow or no growth. Phosphoethanolamine *N*-methyltransferase (NMT) catalyzes the rate-limiting step in tertiary phosphoethanolamine methylation to choline phosphocholine (P-Cho). When P-Cho synthesis was inhibited in the Arabidopsis *peamt* mutant *xipotl*, the plants displayed short primary roots, reduced numbers of root hairs, and abnormal or dead root epidermal cells [41]. Nevertheless, mutant plants supplemented with P-Cho medium reverted to the wild-type phenotype and resumed normal growth, development, and metabolism [41]. Nuccio et al. found that relative to the control, spinach under salt stress exhibited tenfold higher NMT mRNA levels. Thus, *NMT* plays an indispensable role in plant root system development, phospholipid metabolism, and ectodermal cell integrity [42].

Here, we observed that the upregulation of phosphoethanolamine *N*-methyltransferase [E2.1.1.103] (gene-LOC110683223, gene-LOC110730138, NMT) affected choline phosphate accumulation. Therefore, phosphoethanolamine methyltransferase plays a key role in the growth and development of quinoa seedlings under low-phosphorus stress. UDP-sulfoquinovose synthase [EC:3.13.1.1] (gene-LOC110705433, SQD1) and sulfoquinovosyltransferase [EC:2.4.1.-] (gene-LOC110710130, gene-LOC110724176, SQD2) were significantly upregulated in quinoa seedlings under low-phosphorus stress. In contrast, the SQDG content did not significantly differ among quinoa seedlings subjected to different phosphorus levels. Hence, SQDG might not play a major role in photosynthesis, growth, or development in higher plants, in general agreement with the study by Yu B. [43]. Glycerophosphodiester phosphodiesterase (GDE1) catalyzes the conversion of glycerophosphodiester to glycerol-3-phosphate (G-3-P) and corresponding small molecules. GDE1 is an important constituent in the phospholipid metabolic pathway. In the present study, glycerophosphodiester phosphodiesterase [EC:3.1.4.46] (gene-LOC110724999, gene-LOC110694671, GDE1) was significantly upregulated in the quinoa seedlings subjected to low phosphorus levels and significantly downregulated in those exposed to high phosphorus concentrations. Thus, GDE1 is involved in the adaptation of quinoa to low-phosphorus stress and plant growth and development under this condition. The genes mentioned above need further functional verification.

## 4. Materials and Methods

### 4.1. Materials and Sample Preparation

Red quinoa (Dianli-1299) and white quinoa (Dianli-71) were independently selected by Yunnan Agricultural University and planted in Xundian County, Kunming, China (102°41′ E, 25°20′ N). Homogeneous seeds were uniformly sown in pots (117 × 39 × 65 cm) containing substrates with one of three different P_2_O_5_ concentrations (0 kg/hm^2^, 112.5 kg/hm^2^, or 337.5 kg/hm^2^). To each substrate, 112.5 kg/hm^2^ CH_4_N_2_O and K_2_O were also applied. Each pot contained ~500 seedlings that were managed in the early stages according to conventional cultivation techniques, namely, average temperature = 25.6 °C, sunshine duration ~10 h, sowing depth = 2–3 cm, and substrate CH_4_N_2_O, P_2_O_5_, and K_2_O content = 2.75, 1.66, and 1.18 g/kg, respectively. The fertilization was initiated at the two-leaf stage. After 30 d fertilization, the quinoa seedlings grew normally at P_2_O_5_ = 112.5 kg/hm^2^, vigorously at P_2_O_5_ = 37.5 kg/hm^2^, and not at all at P_2_O_5_ = 0 kg/hm^2^. The phenotypic differences were greatest among treatments at this stage. Hence, 30 d after fertilization onset was considered the optimal sampling time, and the metabolome and transcriptome analyses were performed on the aboveground parts of quinoa seedlings at that point (Wuhan MetWare Biotechnology Co. Ltd, Wuhan, China. https://www.metware.cn, accessed on 18 April 2021). Eighteen samples were collected at the same time point on the same day. On the sampling day, rainfall = 0.0 and average temperature = 25.5 °C. Three biological and technical replicates were used. Here, ‘R’ represents red quinoa, ‘W’ represents white quinoa, ‘2’ indicates P_2_O_5_ = 112.5 kg/hm^2^ (CK), ‘4’ indicates P_2_O_5_ = 0 kg/hm^2^ (LP), and ‘5’ indicates P_2_O_5_ = 337.5 kg/hm^2^ (HP). CK includes W2 and R2, LP includes W4 and R4, and HP includes W5 and R5.

### 4.2. Morphological Data Acquisition

After 30 d fertilization, the shoots of the quinoa seedlings were sampled in triplicate, and the plant heights and leaf areas were measured. The plant heights were measured with a vernier caliper from the base to the tip of the uppermost spreading leaf. The leaf areas were measured with a TPYX-A crop leaf morphometer (Zhejiang, China, https://www.tpyn.net, accessed on 18 April 2021).

### 4.3. Metabolite Extraction Detection and Qualitative and Quantitative Analyses

All samples were vacuum freeze-dried (Scientz-100F; Ningbo Scientz Biotechnology Co. Ltd., Zhejiang, China) and pulverized with a grinder (MM400; Retsch GmbH, Haan, Germany). The samples were then extracted in methanol and centrifuged (12,000 rpm, 10 min, 4 °C) to obtain supernatants for the UP-LCMS/MS analysis. The data acquisition system comprised ultra-performance liquid chromatography (SHIMADZU Nexera X2; https://www.shimadzu.com.cn/, accessed on 15 June 2021) and tandem mass spectrometry (Applied Biosystems 4500 QTRAP; http://www.appliedbiosystems.com.cn/, accessed on 15 June 2021) [44]. The substances in the extracts were characterized according to the MetWare database (MWDB; http://en.metware.cn/list/27.html, accessed on 15 June 2021) and by secondary mass spectrometry. The metabolites were quantified by QQQ-MSin MRM. The peak areas were then integrated, and integral corrections were performed [45]. Quality control (QC) samples were prepared by mixing sample extracts. During instrumental analysis, one QC sample was inserted every ten test samples to monitor the repeatability of the analytical process. Total ion flow diagrams (TIC) of the various QC samples were overlapped and analyzed to assess metabolite extraction and detection repeatability. A multivariate statistical analysis was conducted to retain the original data to the fullest extent. A digital model was established after data simplification and dimensionality reduction. The built-in statistical prcomp function in R (www.r-project.org/, accessed on 22 September 2021) was used to plot the analyses between groups and the differences between sample groups [46,47]. A heat map was plotted using the pheatmap package in R. Metabolite accumulation among the various samples was subjected to hierarchical cluster analysis. An orthogonal projection to latent structures discriminant analysis (OPLS-DA) was used to extract the components of the independent variable X and the dependent variable Y and screen for differential variables [48,49]. An OPLS-DA model was obtained based on the results of the OPLS-DA and variable importance in projection (VIP) of the multivariable analysis. The *p*-values and fold changes were combined to screen DEMs [48]. The significantly differentially expressed metabolites between groups were screened for further analysis according to the criteria VIP ≥ 1 and fold change ≥2 or ≤0.5. The DEMs were screened and annotated in the KEGG compound database (http://www.kegg.jp/kegg/compound/, accessed on 22 September 2021). The annotated metabolites were mapped with the KEGG pathway database (http://www.kegg.jp/kegg/pathway.html, accessed on 22 September 2021) [50].

### 4.4. Transcriptome Sequencing and Data Analysis

RNA extraction and detection, library construction, sequencing, and bioinformatics analysis were performed at Beijing Novogene Bioinformatics Technology Co. Ltd., Beijing, China. The total starting RNA was ≥1 μg, and an Illumina NEBNext^®^ UltraTM RNA Library Prep Kit (Illumina, San Diego, CA, USA) was used. After library construction, a Qubit 2.0 fluorometer (Thermo Fisher Scientific, Waltham, MA, USA) was used for preliminary quantification. The library was diluted to 1.5 ng/μL, and an Agilent 2100 Bioanalyzer (Agilent Technologies, Santa Clara, CA, USA) was used to measure the library insert size. If the latter was adequate, qRT-PCR was then performed to quantify the effective concentration (>2 nM) and validate the library quality. Various libraries were pooled according to requirements and used in Illumina sequencing. Fastp v. 0.19.3 (https://github.com/OpenGene/fastp, accessed on 4 July 2021) was used to filter the offline data and remove reads with adapters to obtain clean reads. The latter were then compared against the reference genome (https://www.ncbi.nlm.nih.gov/genome/?Term=Chenopodium+quinoa+Willd, accessed on 3 November 2021) to obtain location data for the latter or the genes as well as unique sample sequence feature information to obtain mapped data. The gene expression levels were quantified and compared with featurecounts v. 1.6.2 in R, and the FPKM (fragments per kilobase of transcript per million fragments mapped) was calculated for each gene based on its length. Differential expression analyses between group pairs were conducted in DESeq2 v. 1.22.1 (https://bioconductor.org/packages/release/bioc/html/DESeq2.html, accessed on 3 November 2021). The total, upregulated, and downregulated DEGs were enumerated for each group. The corrected *p*-values and |log2foldchange| were used as thresholds for significant differential gene expression. The DEGs were functionally annotated with the KEGG (Kyoto Encyclopedia of Genes and Genomes), GO (Gene Ontology), KOG (Karyotic Orthologous Groups), PfAM, Swiss-Prot, TrEMBL, and NR databases.

### 4.5. Combined Transcriptome and Metabolome Analyses

Based on the results of DEM and DEG analyses, those corresponding to the same treatments were simultaneously mapped to the KEGG pathway to clarify the relationships between the genes and the metabolites. According to the results of the DEM and DEG enrichment analyses, histograms were plotted to highlight relative differences in metabolite and gene pathway enrichment. Correlation analyses were performed on the genes and metabolites detected in each differential subgroup. Pearson’s correlation coefficients for the genes and metabolites were calculated with the cor program in R. Nine-quadrant plots were used to show the differential multiplicity of the gene metabolites with Pearson’s correlation coefficients >0.8 in each differential subgroup. All DEMs and DEGs were selected to build an O2PLS (two-way orthogonal projection to latent structures) model. Variables in the different datasets with higher correlations and weights were initially determined by loading plots to filter out the important variables affecting the other omics [51].

### 4.6. RT-qPCR

RNA extracted from the shoots of Dianli-1299 and Dianli-71 were used in RT-qPCR, and the latter was performed in triplicate. The PCR primers were designed with BeaconDesign v. 7.9 (https://beacon-designer.software.informer.com/7.9/, accessed on 19 January 2022) (Table S8). The internal reference gene was *TUB*-*6*. The reagent was PerfectStartTM SYBR qPCR Supermix (TransGen Biotech, Beijing, China). The PCR instrument was ABI Prism7500 (Applied Biosystems, Foster City, CA, USA). The 2^−ΔΔCt^ method was used to analyze the normalized expression of each sample [52].

### 4.7. Statistical Analysis

Microsoft Office 2016 (Microsoft, Washington DC, USA) was used for seedling heights and leaf areas analyses and data plotting. Unsupervised PCA (principal component analysis) was performed by statistics function prcomp within R (www.r-project.org, accessed on 25 January 2022). The data was unit variance scaled before unsupervised PCA. Both HCA and PCC were carried out by R package pheatmap. For HCA, normalized signal intensities of metabolites (unit variance scaling) are visualized as a color spectrum. Significantly regulated metabolites between groups were determined by VIP ≥1 and absolute log2FC (fold change) ≥1. The VIP values were extracted from the OPLS-DA result, which also contains score plots and permutation plots were generated using R package MetaboAnalystR. We used fastp v 0.19.3 to filter the original data, download the reference genome, and its annotation files from the designated website, and we used HISAT v2.1.0 to construct the index and compare clean reads to the reference genome. We used featureCounts v1.6.2 to calculate the gene alignment and then calculate the FPKM of each gene based on the gene length. DESeq2 v1.22.1 was used to analyze the differential expression between the two groups, and the *p*-value was corrected using the Benjamini and Hochberg method. The corrected *p*-value and |log2foldchange| were used as the threshold for significant difference expression. The enrichment analysis is performed based on the hypergeometric test. For KEGG, the hypergeometric distribution test is performed with the unit of the pathway. We used gsea-3.0.jar for the gene setenrichment analysis.

## 5. Conclusions

We analyzed the morphology, metabolomes, and transcriptomes of quinoa seedlings subjected to different phosphorus levels. The latter significantly influenced the phenotypic traits of the quinoa seedlings. The plants under the low-phosphorus treatment were shorter and had smaller leaves than the control, whereas those exposed to moderately high phosphorus levels grew more vigorously, were taller, and had larger leaves than the control. We found that there was no high phosphorus poisoning in high-phosphorus treatment, and we will continue to increase the phosphorus level in the follow-up test. We detected a total of 1057 metabolites, of which those related to glycerophospholipid metabolism, glycerolipid metabolism, and glycolysis and glyconeogenesis were differentially expressed in response to variations in the phosphorus level. We also annotated 29,232 via KEGG transcriptomics. The present study revealed 15 DEMs and 11 DEGs that putatively enable quinoa seedlings to contend with low exogenous phosphorus bioavailability. The findings of this study will help breeders develop quinoa cultivars that tolerate phosphorus deficiency, produce high grain yield and quality, reduce phosphate fertilizer application and costs, and help maintain global food security.

## Figures and Tables

**Table 1:** DEGs in the different treatment groups.

Sample Comparisons	Total No. of Significantly Differentially Expressed Genes (DEGs)	Total No. of Significantly Upregulated DEGs	Total No. of Significantly Downregulated DEGs
R2 vs. R4	2073	1651	422
R2 vs. R5	384	179	205
R4 vs. R5	4019	1524	2495
W2 vs. R2	630	272	358
W2 vs. R4	843	453	390
W2 vs. R5	49	43	6
W4 vs. R4	3191	1976	1215
W4 vs. W5	1489	679	810
W5 vs. R4	177	117	60

## Data Availability

Not applicable.

## Supplementary Materials

The following supporting information can be downloaded at: https://www.mdpi.com/article/10.3390/ijms23094704/s1.
