# Supplementary material for: Transcriptome and Metabolome Analyses Revealed the Response Mechanism of Quinoa Seedlings to Different Phosphorus Stresses
Source: Int J Mol Sci. 2022 Apr 24;23(9):4704. doi: 10.3390/ijms23094704 (PMC9105174; doi:10.3390/ijms23094704)
Supplement: Supplementary file 1 [file ijms-23-04704-s001.zip › Figure.S1.pdf]

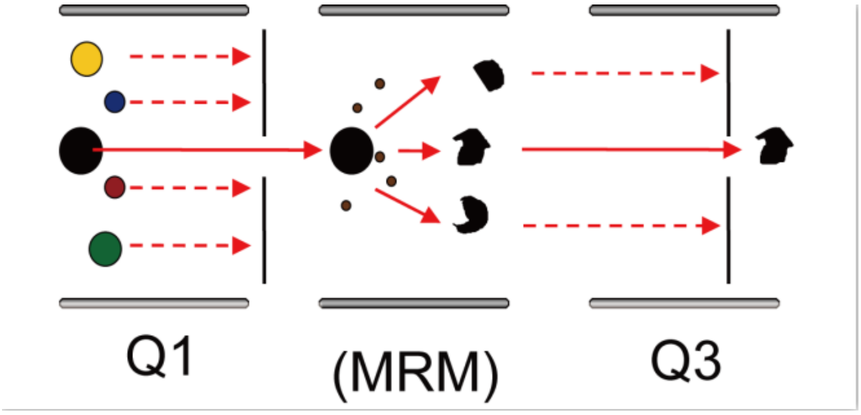

Figure S1. In MRM mode, the four-stage rod eliminates interference by screening the precursor (parent) ions of the target substance and excluding those corresponding to substances with other MW. The precursor ions are induced and ionized by the collision chamber and then break into fragment ions. The latter are then filtered through the triple four-stage rod to select the required characteristic fragment ion and eliminate interference from non-target ions. This procedure improves quantification accuracy and repeatability.
