# Supplementary material for: Transcriptome and Metabolome Analyses Revealed the Response Mechanism of Quinoa Seedlings to Different Phosphorus Stresses
Source: Int J Mol Sci. 2022 Apr 24;23(9):4704. doi: 10.3390/ijms23094704 (PMC9105174; doi:10.3390/ijms23094704)
Supplement: Supplementary file 1 [file ijms-23-04704-s001.zip › Figure.S10.pdf]

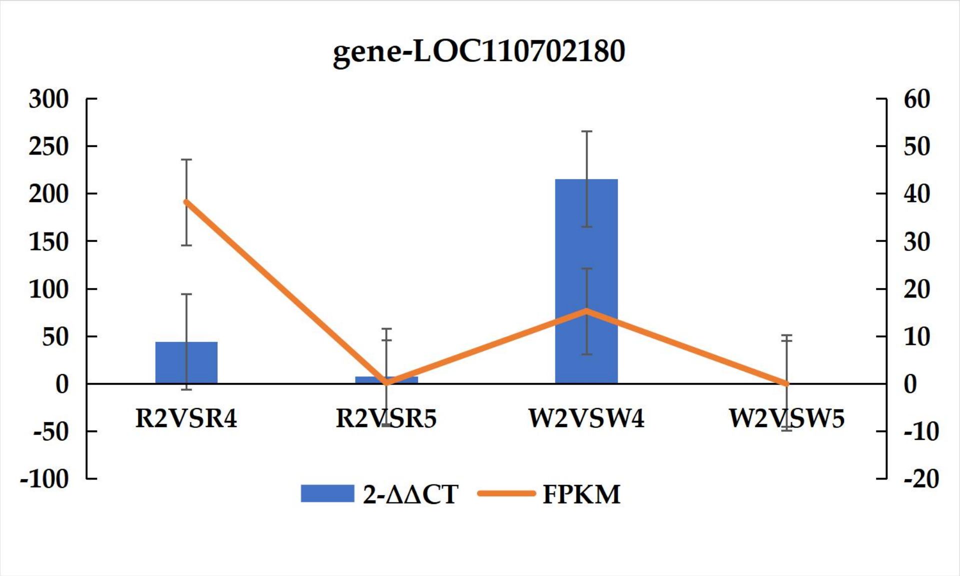
gene-LOC110702180
300
250
200
150
100
50
0
-50
-100
60
50
40
30
20
10
0
-10
-20
R2VSR4
R2VSR5
W2VSW4
W2VSW5
2-ΔΔCT
FPKM

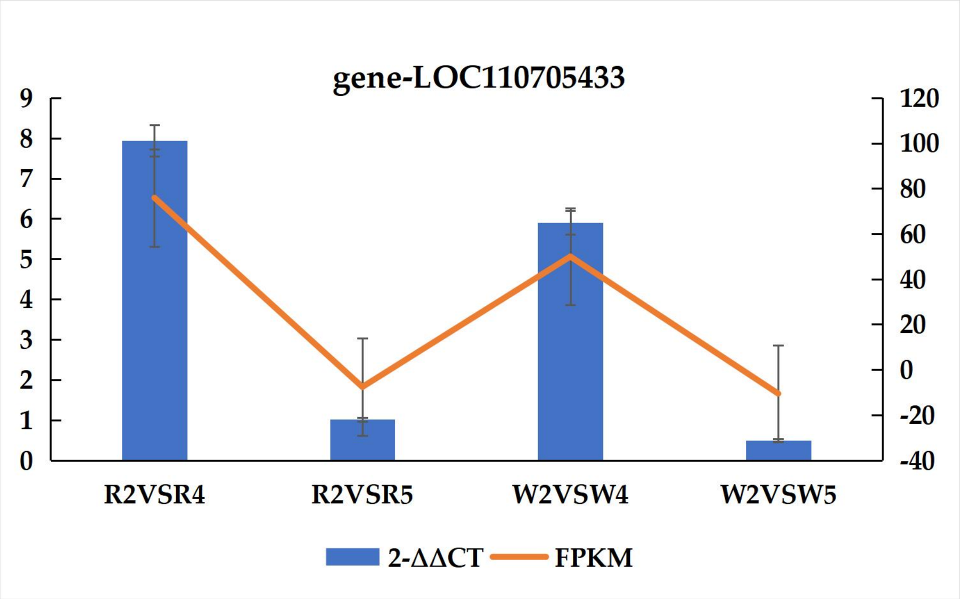
gene-LOC110705433
9
8
7
6
5
4
3
2
1
0
120
100
80
60
40
20
0
-20
-40
R2VSR4
R2VSR5
W2VSW4
W2VSW5
2-ΔΔCT
FPKM

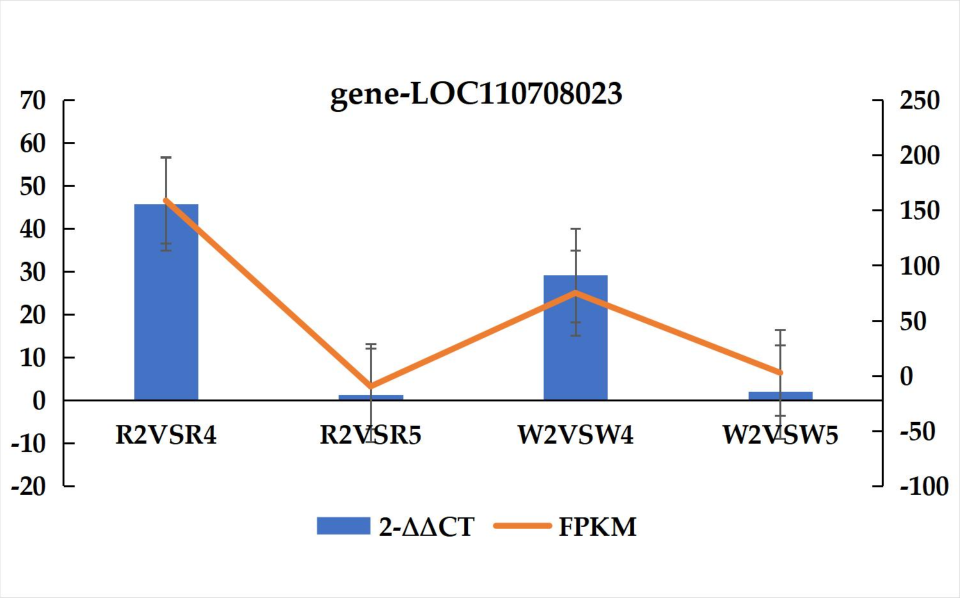
gene-LOC110708023
70
60
50
40
30
20
10
0
-10
-20
250
200
150
100
50
0
-50
-100
R2VSR4
R2VSR5
W2VSW4
W2VSW5
2-ΔΔCT
FPKM

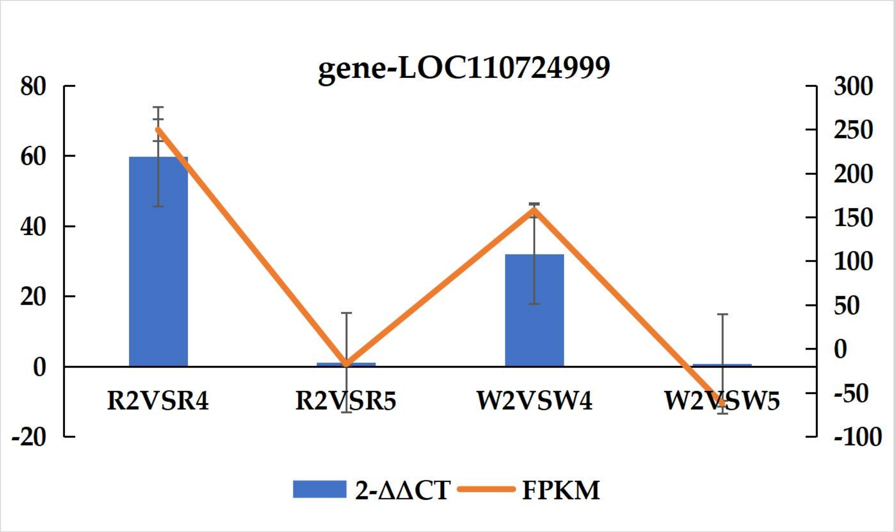
gene-LOC110724999
80
60
40
20
0
-20
300
250
200
150
100
50
0
-50
-100
R2VSR4
R2VSR5
W2VSW4
W2VSW5
2-ΔΔCT
FPKM

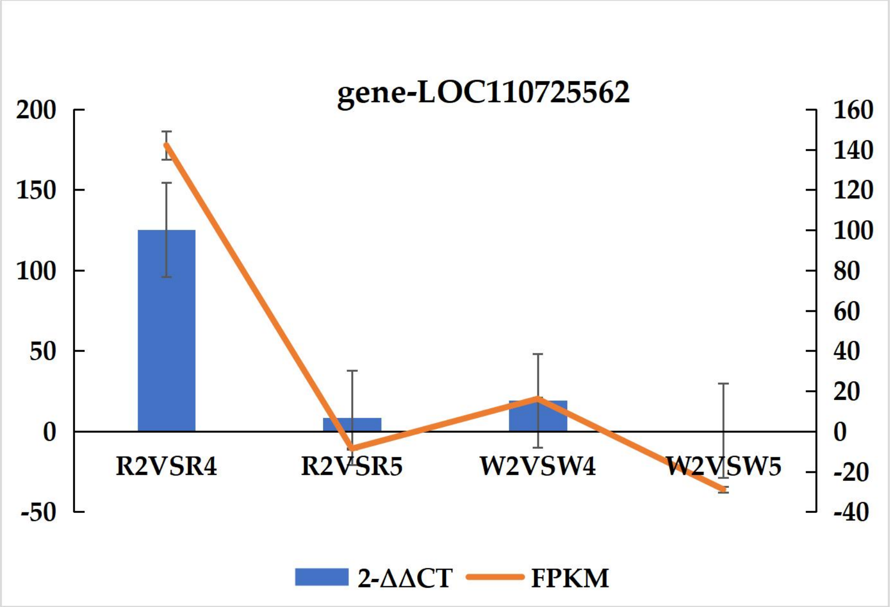
gene-LOC110725562
200
150
100
50
0
-50
160
140
120
100
80
60
40
20
0
-20
-40
R2VSR4
R2VSR5
W2VSW4
W2VSW5
2-ΔΔCT
FPKM

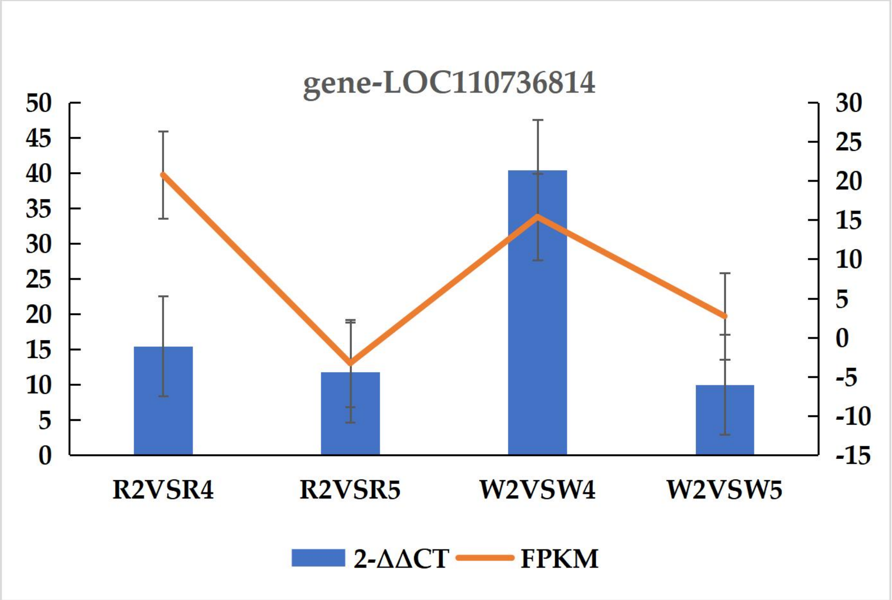
gene-LOC110736814
50
45
40
35
30
25
20
15
10
5
0
30
25
20
15
10
5
0
-5
-10
-15
R2VSR4
R2VSR5
W2VSW4
W2VSW5
2-ΔΔCT
FPKM

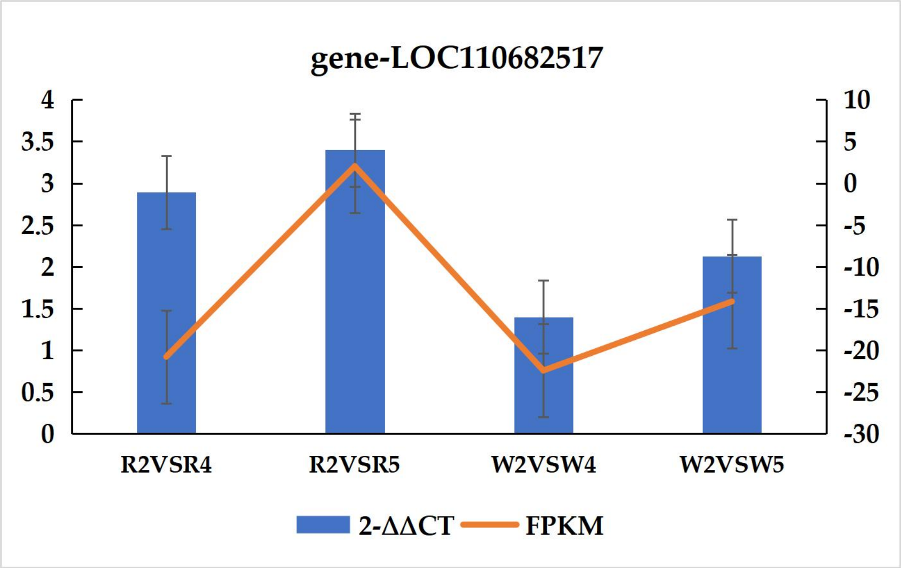


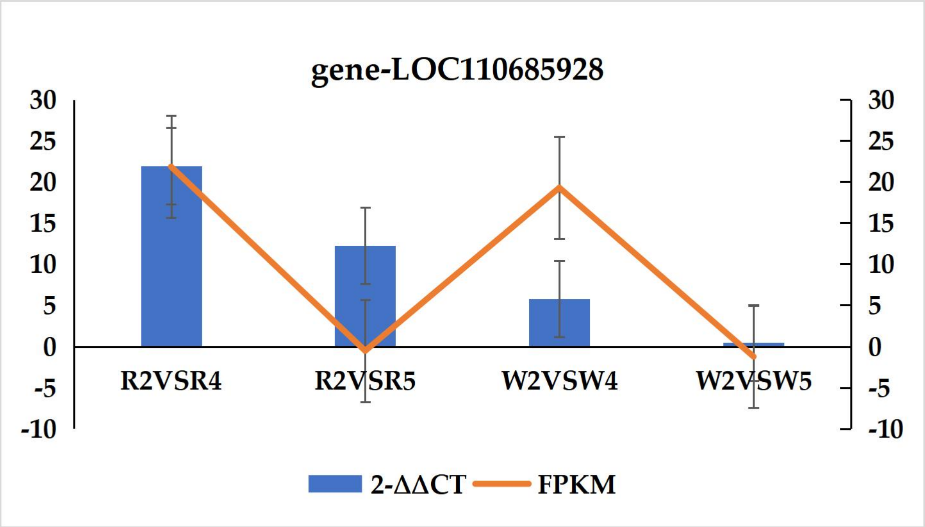


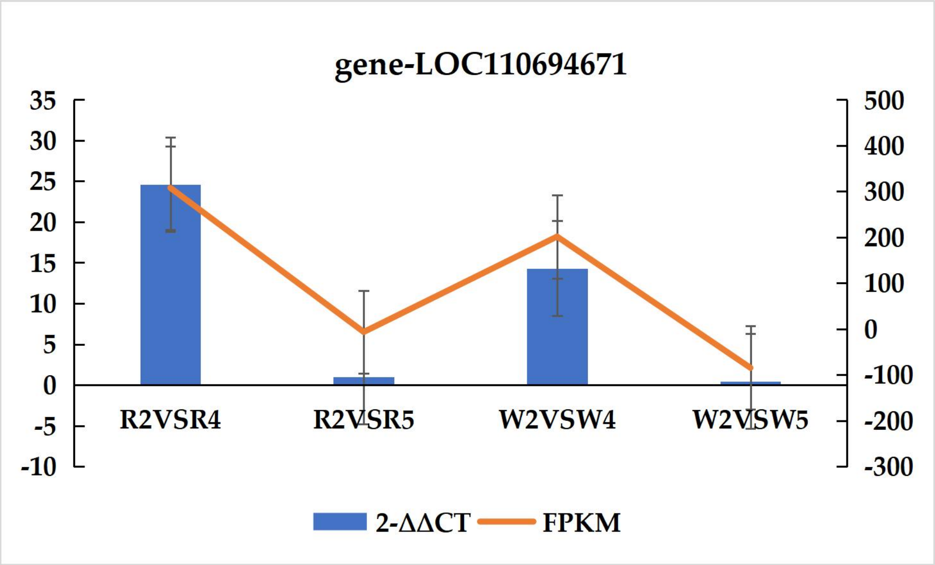


Figure S10. Comparison of RT-qPCR and RNA-Seq results. Histogram: RT-qPCR results. Broken-line diagram: RNA-Seq data. The ordinate represents the expression level. The abscissa represents different groups.
