# Supplementary material for: Transcriptome and Metabolome Analyses Revealed the Response Mechanism of Quinoa Seedlings to Different Phosphorus Stresses
Source: Int J Mol Sci. 2022 Apr 24;23(9):4704. doi: 10.3390/ijms23094704 (PMC9105174; doi:10.3390/ijms23094704)
Supplement: Supplementary file 1 [file ijms-23-04704-s001.zip › Figure.S2.pdf]

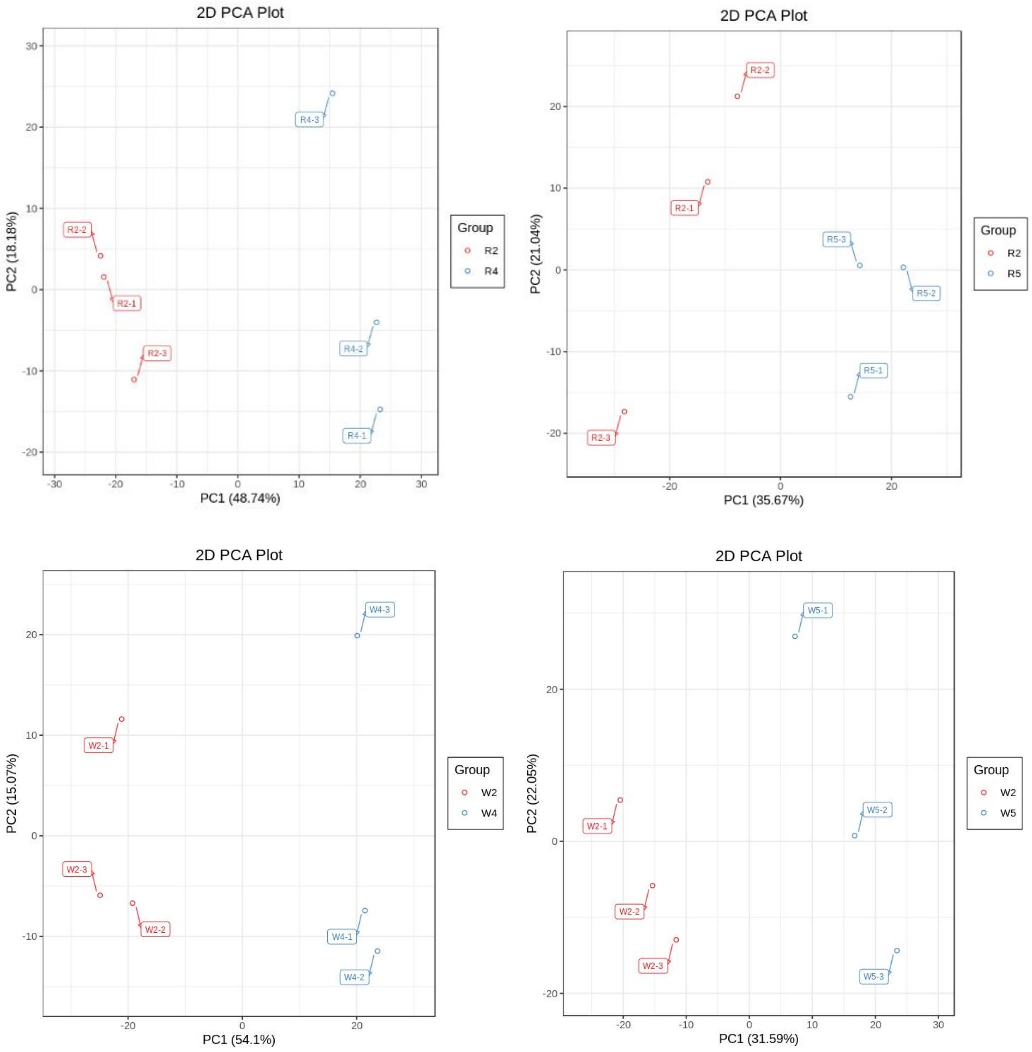

Figure S2. Principal component analyses (PCA) of different groups. Note: each group has a PCA chart. PC1 represents the first principal component. PC2 represents the second principal component. PC3 represents the third principal component. The percentage represents the rate of interpretation of the PC to the dataset. Each point in the figure represents a sample. Samples within the same group are represented by the same color. Each group is a unique variety.
