# Supplementary material for: Transcriptome and Metabolome Analyses Revealed the Response Mechanism of Quinoa Seedlings to Different Phosphorus Stresses
Source: Int J Mol Sci. 2022 Apr 24;23(9):4704. doi: 10.3390/ijms23094704 (PMC9105174; doi:10.3390/ijms23094704)
Supplement: Supplementary file 1 [file ijms-23-04704-s001.zip › Figure.S3.pdf]

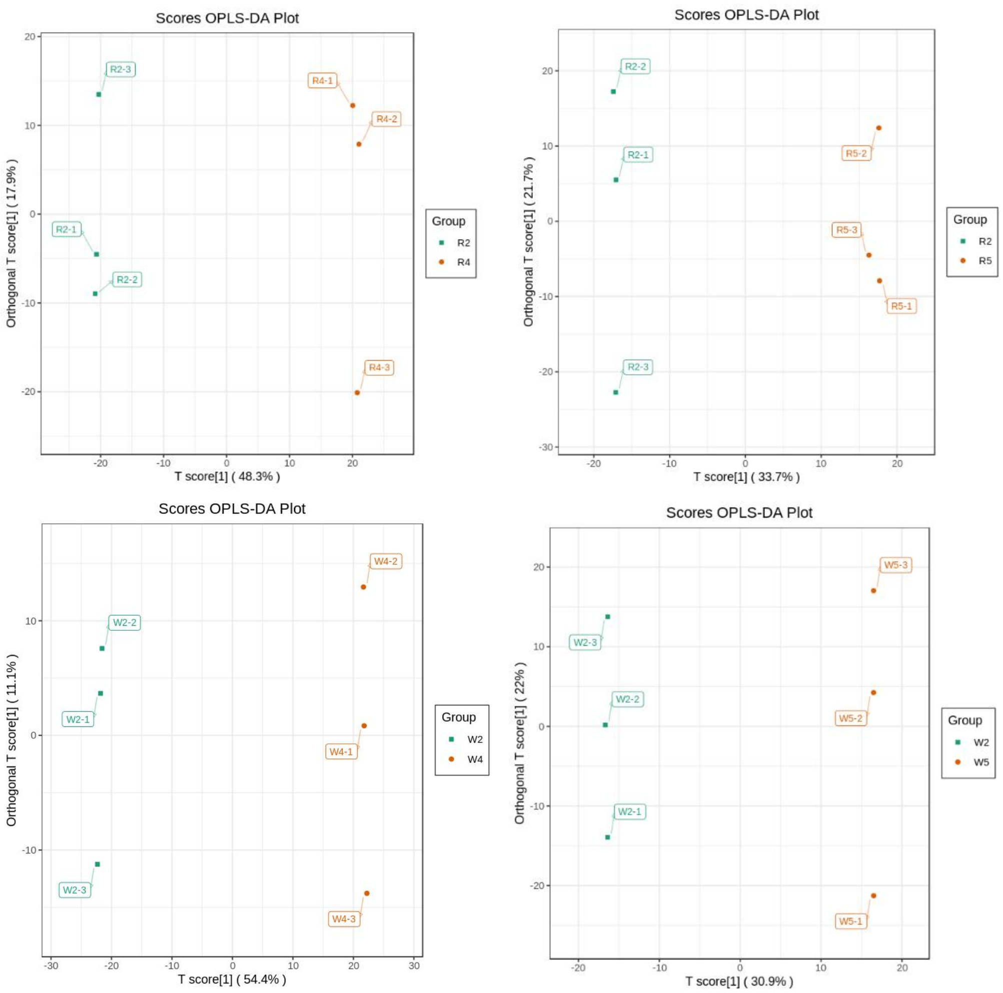


Figure S3. OPLS-DA score chart. Note: the abscissa represents the predicted PC. The differences between groups are in the direction of the abscissa. The ordinate represents the orthogonal PC. The differences within groups are in the direction of the ordinate. The percentage represents the interpretation of the PC to the dataset. Each point in the figure represents a sample. Samples in the same group are represented by the same color. Each group is a unique variety.
