# Supplementary material for: Transcriptome and Metabolome Analyses Revealed the Response Mechanism of Quinoa Seedlings to Different Phosphorus Stresses
Source: Int J Mol Sci. 2022 Apr 24;23(9):4704. doi: 10.3390/ijms23094704 (PMC9105174; doi:10.3390/ijms23094704)
Supplement: Supplementary file 1 [file ijms-23-04704-s001.zip › Figure.S4.pdf]

R2 VS. R4

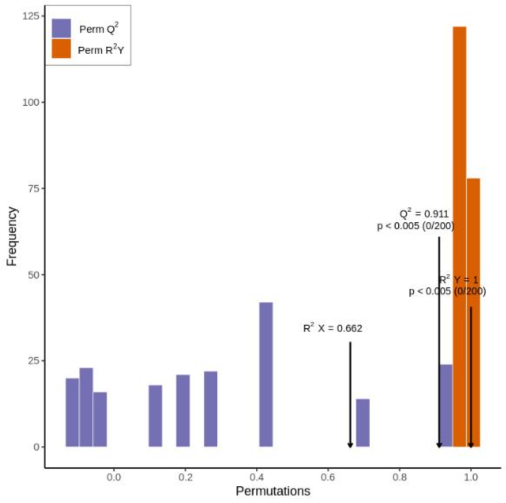

R2 VS. R5

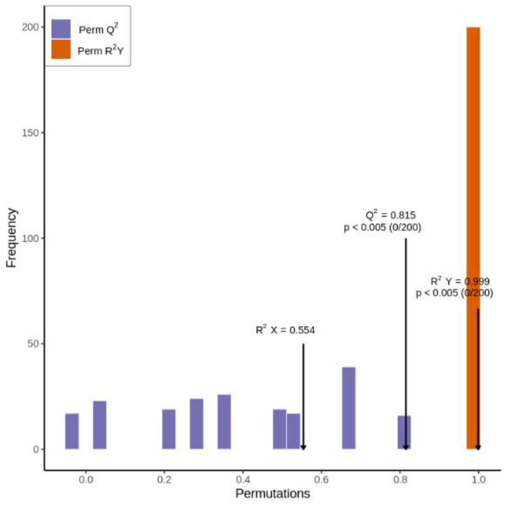

W2 VS. W4

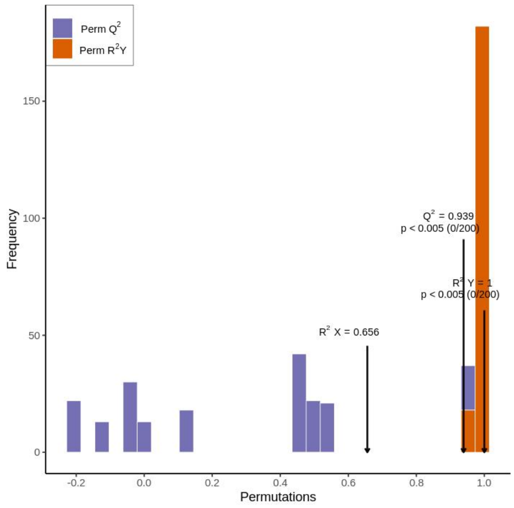

W2 VS. W5

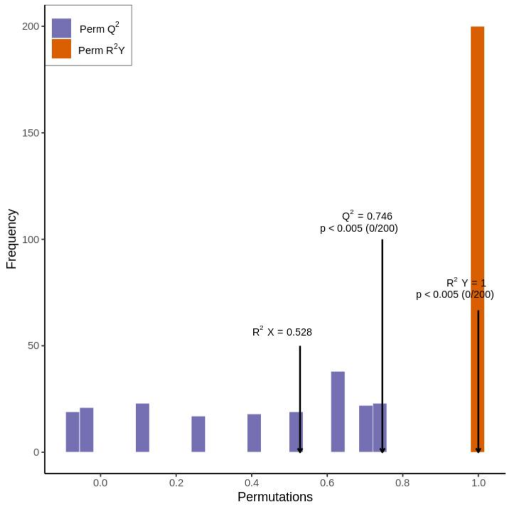

Figure S4. OPLS-DA model validation diagram. The abscissa represents the model accuracy. The ordinate is the model classification effect frequency. The model performs 200 random arrangements and combination experiments on the data. If P = 0.02 for Q^2, the prediction ability of four random grouping models in this permutation test is better than that of the OPLS-DA model. If P = 0.545 for R^2Y, there are 109 random grouping models in the permutation test and their rate of interpretation for the Y matrix is better than that of the OPLS-DA model. In general, the model is optimal at $P < 0.05$.
