# Supplementary material for: Transcriptome and Metabolome Analyses Revealed the Response Mechanism of Quinoa Seedlings to Different Phosphorus Stresses
Source: Int J Mol Sci. 2022 Apr 24;23(9):4704. doi: 10.3390/ijms23094704 (PMC9105174; doi:10.3390/ijms23094704)
Supplement: Supplementary file 1 [file ijms-23-04704-s001.zip › Figure.S5.pdf]

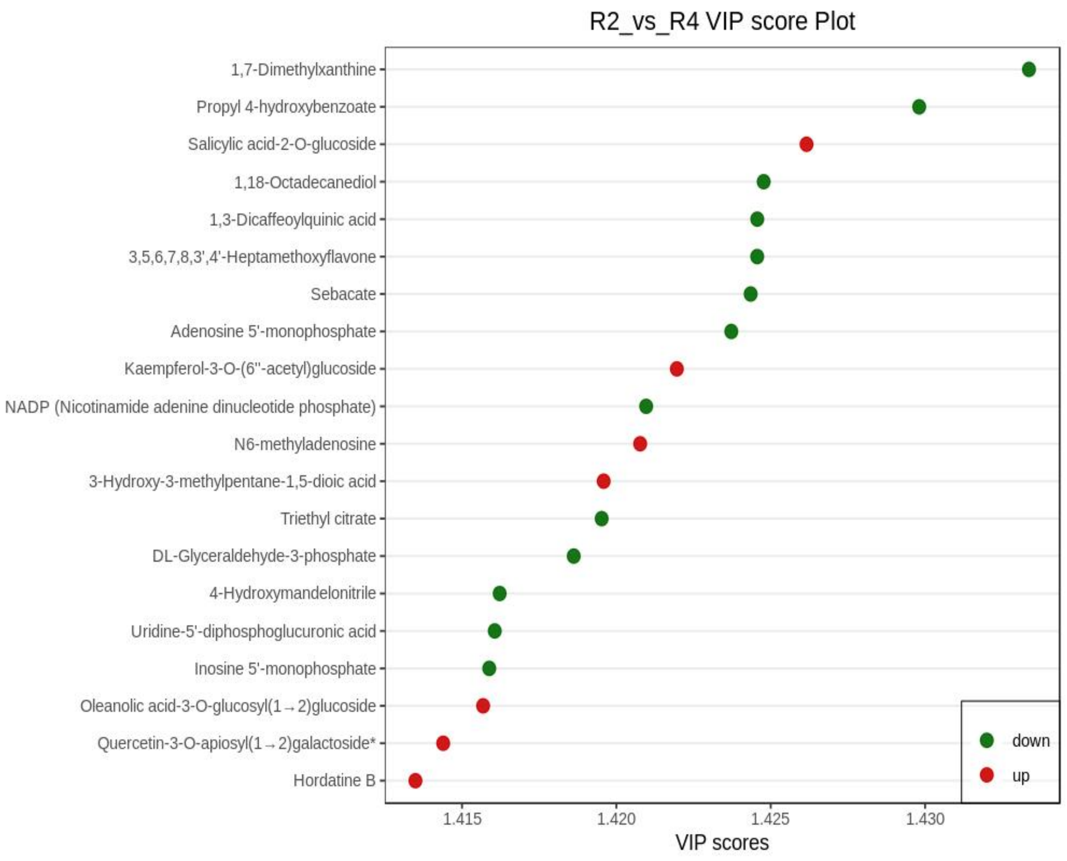

R2_vs_R4 VIP score Plot
1,7-Dimethylxanthine
Propyl 4-hydroxybenzoate
Salicylic acid-2-O-glucoside
1,18-Octadecanediol
1,3-Dicaffeoylquinic acid
3,5,6,7,8,3',4'-Heptamethoxyflavone
Sebacate
Adenosine 5'-monophosphate
Kaempferol-3-O-(6''-acetyl)glucoside
NADP (Nicotinamide adenine dinucleotide phosphate)
N6-methyladenosine
3-Hydroxy-3-methylpentane-1,5-dioic acid
Triethyl citrate
DL-Glyceraldehyde-3-phosphate
4-Hydroxymandelonitrile
Uridine-5'-diphosphoglucuronic acid
Inosine 5'-monophosphate
Oleanolic acid-3-O-glucosyl(1→2)glucoside
Quercetin-3-O-apiosyl(1→2)galactoside*
Hordatine B
down
up
1.415
1.420
1.425
1.430
VIP scores

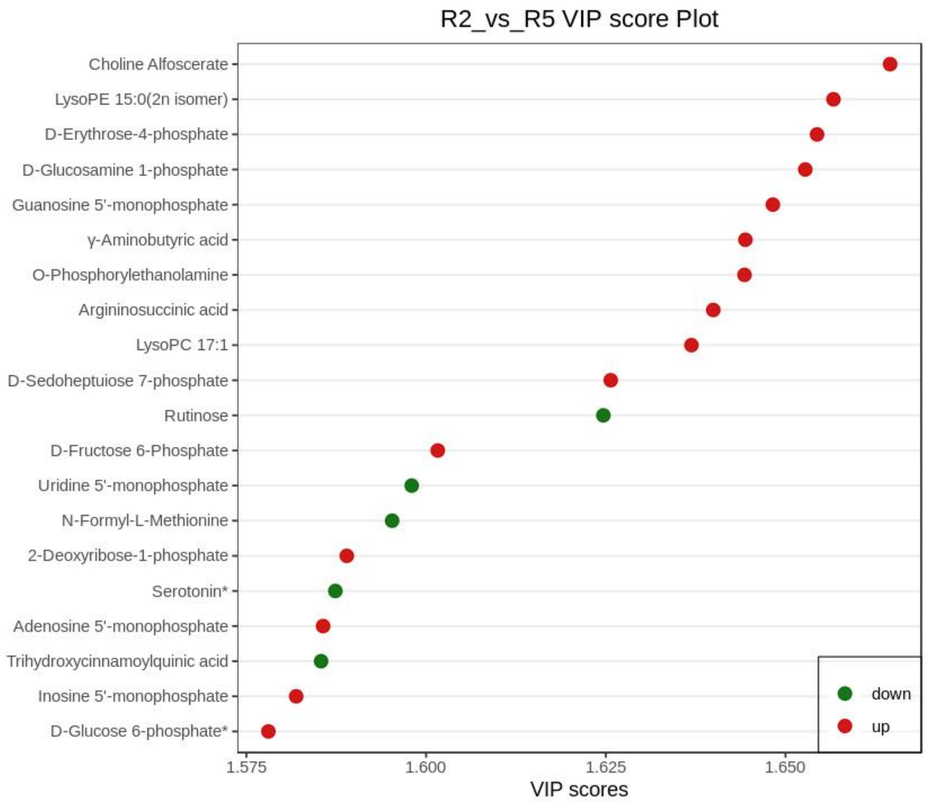

R2_vs_R5 VIP score Plot
Choline Alfoscerate
LysoPE 15:0(2n isomer)
D-Erythrose-4-phosphate
D-Glucosamine 1-phosphate
Guanosine 5'-monophosphate
γ-Aminobutyric acid
O-Phosphorylethanolamine
Argininosuccinic acid
LysoPC 17:1
D-Sedoheptuiose 7-phosphate
Rutinose
D-Fructose 6-Phosphate
Uridine 5'-monophosphate
N-Formyl-L-Methionine
2-Deoxyribose-1-phosphate
Serotonin*
Adenosine 5'-monophosphate
Trihydroxycinnamoylquinic acid
Inosine 5'-monophosphate
D-Glucose 6-phosphate*
down
up
1.575
1.600
1.625
1.650
VIP scores

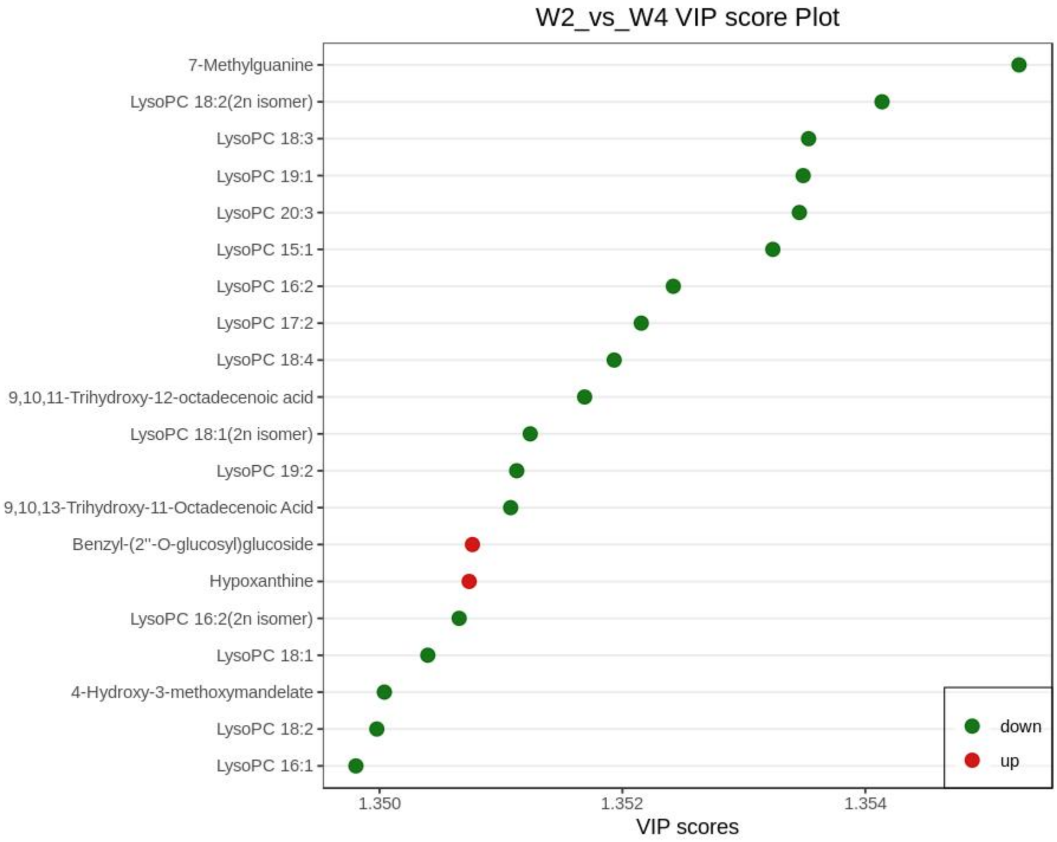

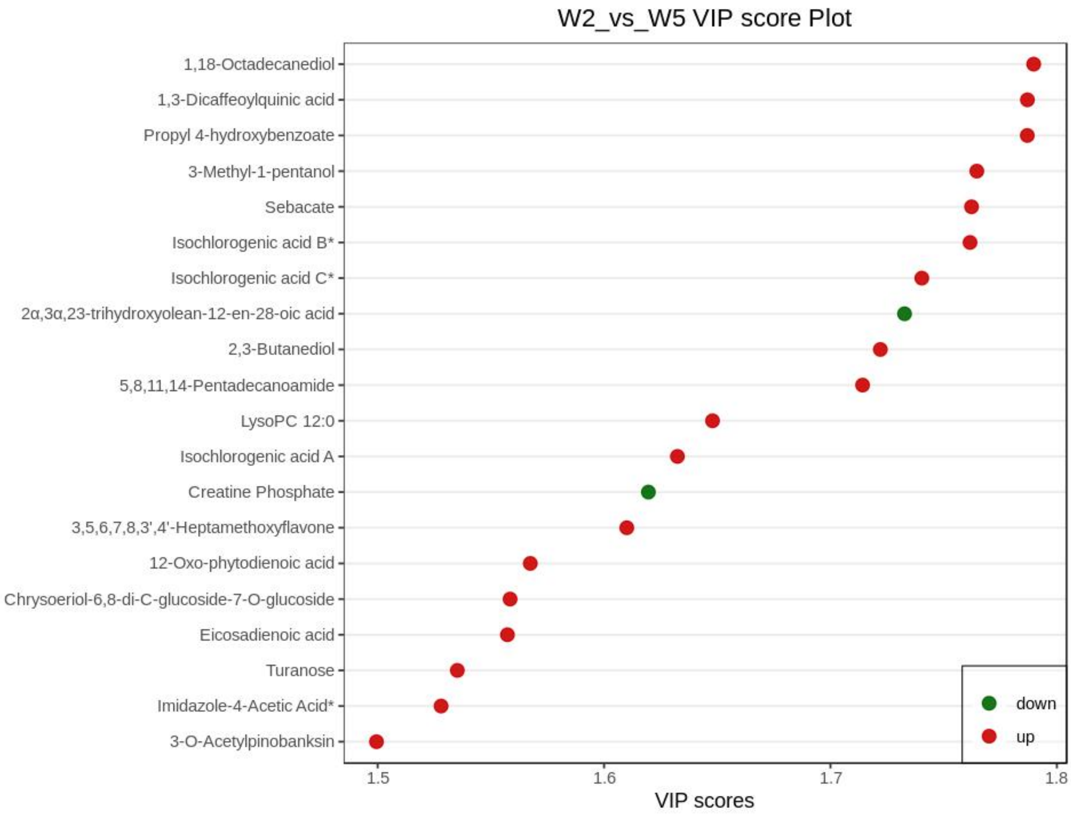

Figure S5. Variable importance projection (VIP) value map of differentially expressed metabolites (DEMs). Note: the abscissa represents the VIP value. The ordinate represents DEMs. Red represents DEM upregulation. Green represents DEM downregulation.
