# Supplementary material for: Transcriptome and Metabolome Analyses Revealed the Response Mechanism of Quinoa Seedlings to Different Phosphorus Stresses
Source: Int J Mol Sci. 2022 Apr 24;23(9):4704. doi: 10.3390/ijms23094704 (PMC9105174; doi:10.3390/ijms23094704)
Supplement: Supplementary file 1 [file ijms-23-04704-s001.zip › Figure.S7.pdf]

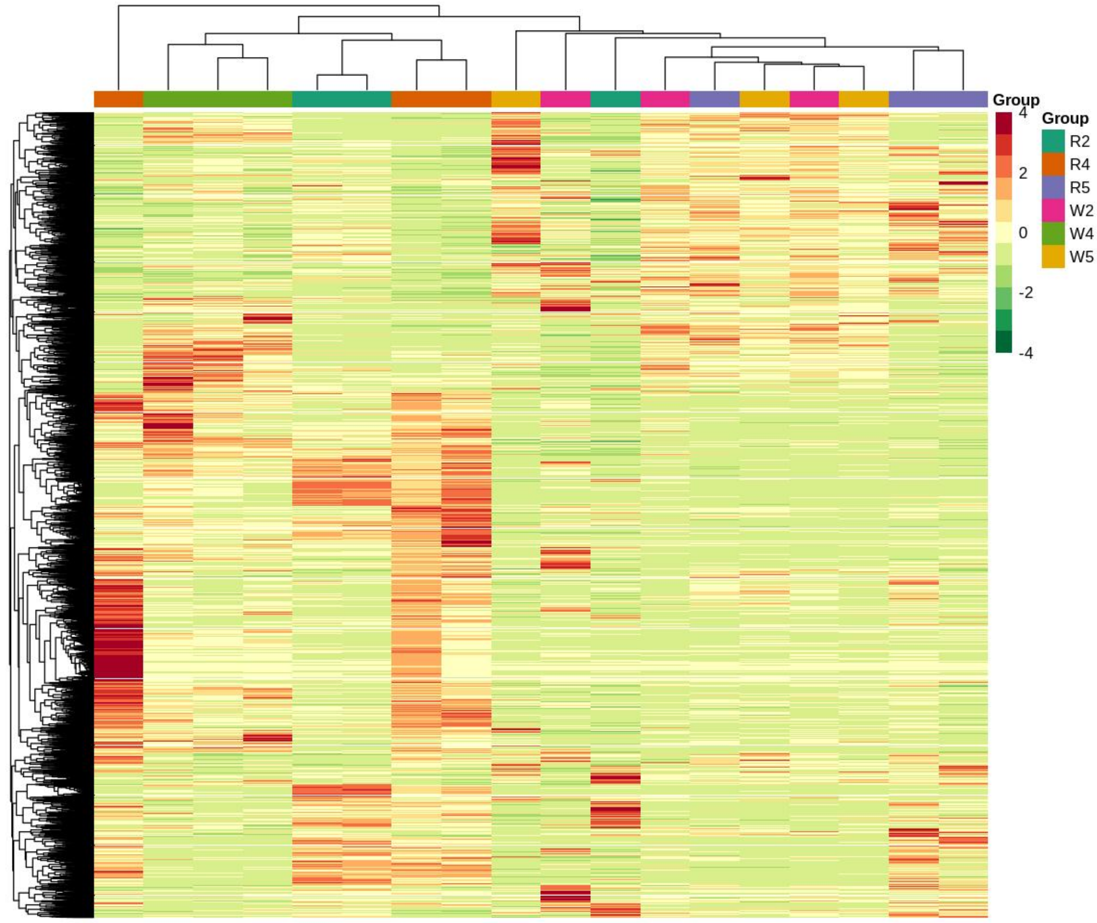

Figure S7. Differentially expressed gene (DEGs) clustering heat map. The abscissa represents the sample name and the hierarchical clustering results. The ordinate represents the DEGs and hierarchical clustering results. Red indicates high expression (up-regulation) and green indicates low expression (down-regulation).
