# Supplementary material for: Transcriptome and Metabolome Analyses Revealed the Response Mechanism of Quinoa Seedlings to Different Phosphorus Stresses
Source: Int J Mol Sci. 2022 Apr 24;23(9):4704. doi: 10.3390/ijms23094704 (PMC9105174; doi:10.3390/ijms23094704)
Supplement: Supplementary file 1 [file ijms-23-04704-s001.zip › Figure.S8.pdf]

R2 vs. R4

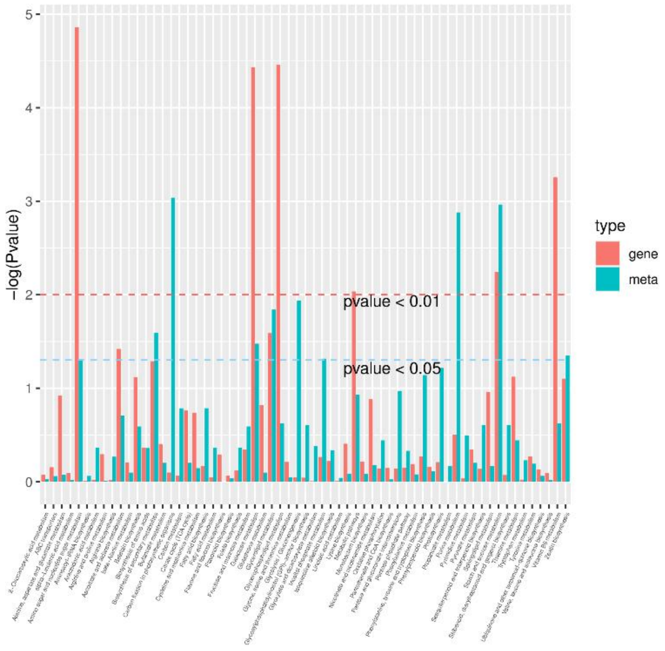


W2 vs. W4

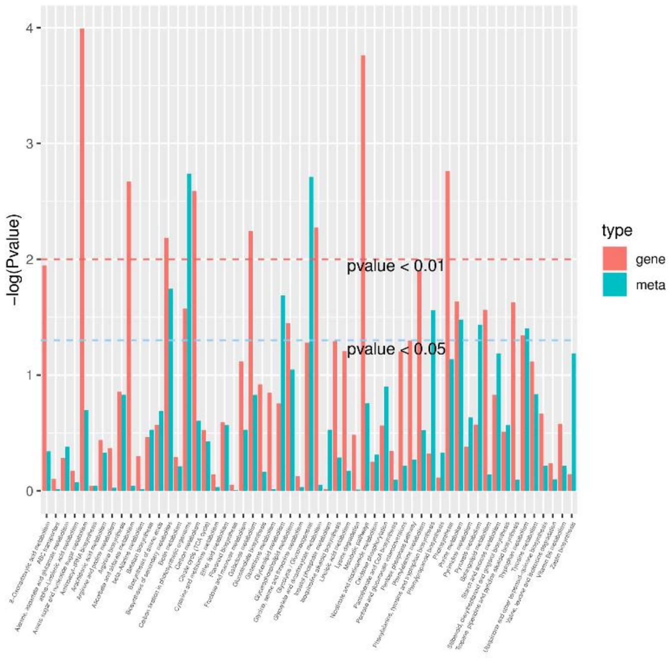


Figure S8. KEGG enrichment analysis P-value histogram. A histogram is plotted to show the degrees of DEM and DEG pathway enrichment based on the enrichment analyses. The abscissa represents the metabolic pathway. Red in the ordinate represents the enriched P-value of the DEGs. Green represents the enriched p-value of the DEMs and is expressed as -log P. The degree of enrichment increases with ordinate height.
