# Supplementary material for: Transcriptome and Metabolome Analyses Revealed the Response Mechanism of Quinoa Seedlings to Different Phosphorus Stresses
Source: Int J Mol Sci. 2022 Apr 24;23(9):4704. doi: 10.3390/ijms23094704 (PMC9105174; doi:10.3390/ijms23094704)
Supplement: Supplementary file 1 [file ijms-23-04704-s001.zip › Figure.S9.pdf]

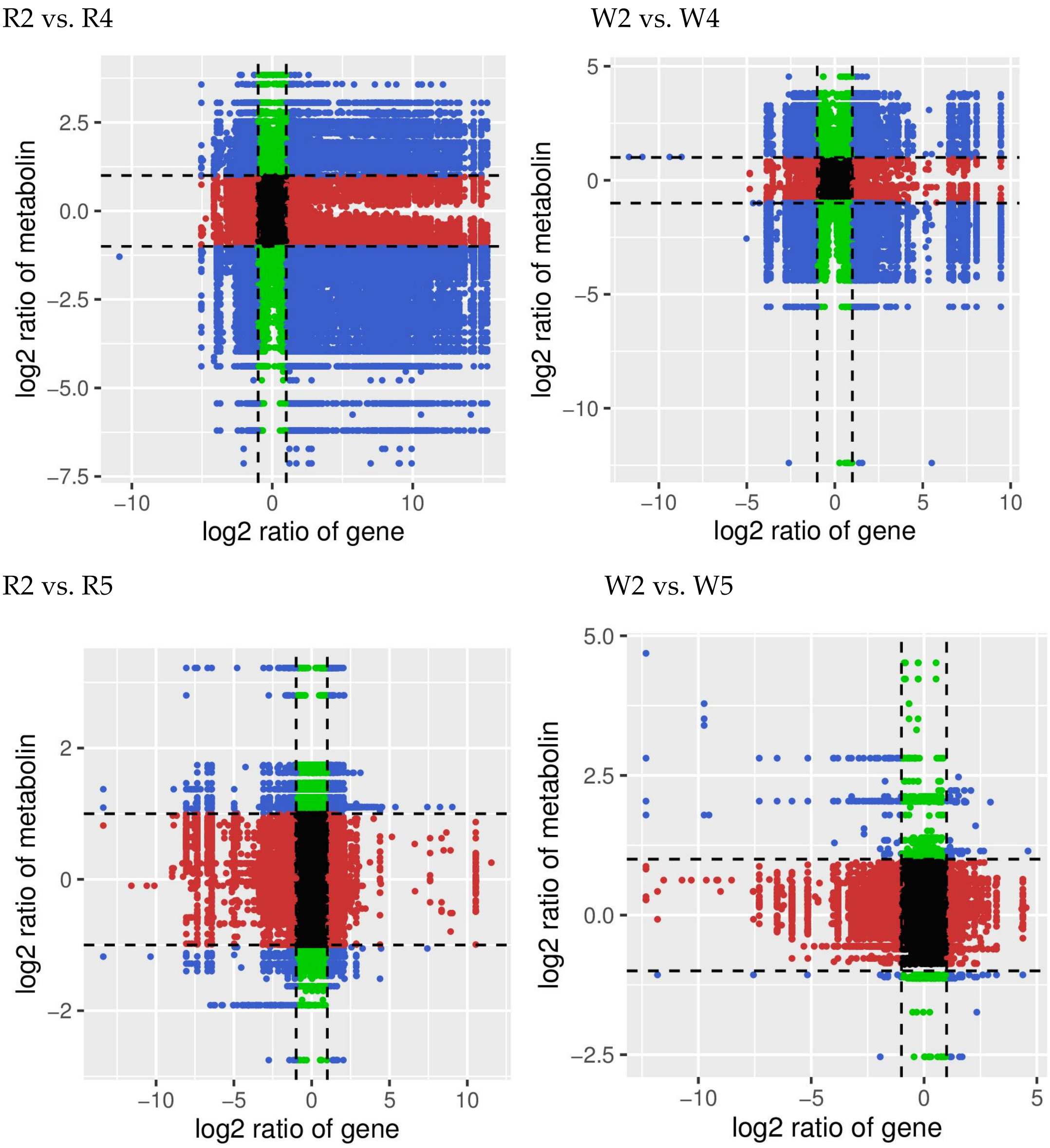

Figure S9. Correlation analysis and nine-quadrant chart. The black dotted line is divided into quadrants 1–9 from left to right and from top to bottom. For quadrant 5, the genes, metabolites, and differently grouped genes and metabolites are not differentially expressed. For quadrants 3 and 7, the genes and metabolites have the same differential expression patterns. For genes and metabolites with consistent expression level trends, the genes may positively regulate the changes in the metabolites. For quadrants 1 and 9, the genes and metabolites have the opposite differential expression patterns. For genes and metabolites with inconsistent expression level trends, the genes may negatively regulate the changes in the metabolites. For quadrants 2, 4, 6, 8, when the metabolites are unchanged, the genes are upregulated or downregulated and when the genes are unchanged, the metabolites are upregulated or downregulated.
